# Unveiling Cas12j Trans‐Cleavage Activity for CRISPR Diagnostics: Application to miRNA Detection in Lung Cancer Diagnosis

**DOI:** 10.1002/advs.202402580

**Published:** 2024-10-01

**Authors:** Ju‐Eun Kang, Hansol Kim, Young‐Hoon Lee, Ha‐Yeong Lee, Yeonkyung Park, Hyowon Jang, Jae‐Rin Kim, Min‐Young Lee, Byeong‐Ho Jeong, Ju‐Young Byun, Seung Jun Kim, Eun‐Kyung Lim, Juyeon Jung, Eui‐Jeon Woo, Taejoon Kang, Kwang‐Hyun Park

**Affiliations:** ^1^ Critical Diseases Diagnostics Convergence Research Center Korea Research Institute of Bioscience and Biotechnology (KRIBB) Daejeon 34141 Republic of Korea; ^2^ Department of Proteome Structural Biology KRIBB School of Bioscience University of Science and Technology (UST) Daejeon 34113 Republic of Korea; ^3^ Bionanotechnology Research Center KRIBB Daejeon 34141 Republic of Korea; ^4^ Department of Nano‐Bio Convergence, Surface Materials Division Korea Institute of Materials Science (KIMS) Changwon Gyeongsangnam‐do 51508 Republic of Korea; ^5^ Division of Pulmonary and Critical Care Medicine Department of Medicine Samsung Medical Center Sungkyunkwan University (SKKU) School of Medicine Seoul 06351 Republic of Korea; ^6^ School of Pharmacy SKKU Suwon Gyeongi‐do 16419 Republic of Korea; ^7^ Department of Nanobiotechnology KRIBB School of Biotechnology, UST Daejeon 34113 Republic of Korea; ^8^ Disease Target Structure Research Center KRIBB Daejeon 34141 Republic of Korea

**Keywords:** Cas12j, CRISPR, liquid biopsy, lung cancer, miRNA

## Abstract

Cas12j, a hypercompact and efficient Cas protein, has potential for use in CRISPR diagnostics, but has not yet been used because the trans‐cleavage activity of Cas12j is veiled. Here, the trans‐cleavage behavior of Cas12j1, 2, and 3 variants and evaluate their suitability for nucleic acid detection is unveiled. The target preferences and mismatch specificities of the Cas12j variants are precisely investigated and the optimal Cas12j reaction conditions are determined. As a result, the EXP‐J assay for miRNA detection by harnessing the robust trans‐cleavage activity of Cas12j on short ssDNA is developed. The EXP‐J method demonstrates exceptional detection capabilities for miRNAs, proving that Cas12j can be a pivotal component in molecular diagnostics. Furthermore, the translational potential of the EXP‐J assay is validated by detecting oncogenic miRNAs in plasma samples from lung cancer patients. This investigation not only elucidates the trans‐cleavage characteristics of Cas12j variants, but also advances the Cas12j‐based diagnostic toolkit.

## Graphical abstract

Here, the trans‐cleavage behavior of Cas12j1, 2, and 3 variants, evaluating their suitability for nucleic acid detection is unveiled. Harnessing the robust trans‐cleavage activity of Cas12j on short ssDNA, the EXP‐J assay for miRNA detection is developed. This investigation not only elucidates the trans‐cleavage characteristics of Cas12j variants, but also advances the Cas12j‐based diagnostic toolkit.

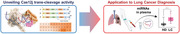

## 1 Introduction

The clustered regularly interspaced short palindromic repeats (CRISPR) and CRISPR‐associated proteins (Cas) systems, originally derived from microbial adaptive immune mechanisms, have initiated a paradigm shift in the fields of genome editing and molecular detection.^[^ ^1^ ^]^ These systems can be divided into two main classes: Class 1 and Class 2. Class 1 is further subdivided into Type I, III, and IV systems, while Class 2 diverges into Type II (e.g., Cas9), V (e.g., Cas12), and VI (e.g., Cas13) systems.^[^ ^2^ ^]^ Class 1 systems function through multi‐protein complexes, while Class 2 systems use a single effector protein in conjunction with CRISPR RNA (crRNA) to form a ribonucleoprotein (RNP) complex capable of recognizing and cleaving specific sequences.^[^ ^3^ ^]^ A notable property shared by several Class 2 system effectors such as Cas12a, Cas12b, Cas12f1, and Cas13a is collateral cleavage, often referred to as trans‐cleavage.^[^ ^4^ ^]^ When the crRNA within the Cas protein accurately binds to its target sequences, precise on‐target cleavage (cis‐cleavage) occurs. The Cas/crRNA complex then indiscriminately cleaves nearby single‐stranded nucleic acids (trans‐cleavage). This unique activity of Class 2 effectors paves the way for the development of a variety of CRISPR‐based diagnostic methods such as specific high‐sensitivity enzymatic reporter unlocking (SHERLOCK), DNA endonuclease‐targeted CRISPR trans reporter (DETECTR), combinatorial arrayed reactions for multiplexed evaluation of nucleic acids (CARMEN), and CRISPR‐based amplification‐free digital RNA detection (SATORI), among others,^[^ ^4^, ^5^ ^]^ contributing to the fields of disease diagnosis and prognosis, food safety testing, environmental monitoring, and more.^[^ ^6^ ^]^

Among the Class 2 CRISPR systems, Type V systems encompass a wide range of subtypes from Type V‐A to Type V‐K, each characterized by a unique set of Cas12 effectors.^[^ ^2^ ^]^ Cas12j, also known as CasΦ, is one of the effector proteins within the Type V CRISPR system.^[^ ^7^ ^]^ It is encoded within the Biggiephage clade, which distinguishes it from other Type V effectors. Despite sharing less than 7% amino acid identity with other Type V CRISPR effectors, Cas12j has the ability to recognize the protospacer adjacent motif (PAM) at the 3′ end of target sequences and initiate staggered cuts on both target and non‐target DNA strands.^[^ ^8^ ^]^ Therefore, Cas12j has the potential to serve as a versatile tool in the biotechnology arena, similar to other established CRISPR systems. In particular, Cas12j is smaller than other Cas proteins, with only 700 to 800 residues, making it a compact and efficient effector for diagnostic applications.^[^ ^9^ ^]^ However, Cas12j has not yet been used in CRISPR diagnostics because its unique operational properties remain underexplored. There is a gray area in understanding its trans‐cleavage efficiency, a key metric for the performance of diagnostic tools. In this context, we aimed to elucidate the trans‐cleavage activity of Cas12j variants and their potential suitability for nucleic acid detection.

Herein, we unveiled the trans‐cleavage activity of three Cas12j variants (Cas12j1, 2, and 3) and explored their applicability in nucleic acid detection. The investigation was carried out through carefully designed experiments that addressed different facets of trans‐cleavage efficiency and specificity, which are critical for the optimization of Cas12j‐based diagnostic platforms. First, we extensively investigated the buffer and reaction conditions that could potentiate the trans‐cleavage activity of Cas12j variants. Through rigorous testing under varying pH, salt concentration, cofactor composition, temperature, Cas/crRNA ratio, and target nucleic acid type, we identified the optimal conditions that significantly enhanced the trans‐cleavage efficiency of these variants. We then exploited the enhanced trans‐cleavage activity of Cas12j for the detection of microRNAs (miRNAs), key regulators of gene expression with profound implications in numerous biological processes and diseases.^[^ ^10^ ^]^ The detection system integrating the exponential amplification reaction (EXPAR) with Cas12j was developed to facilitate the amplification and subsequent detection of target miRNAs. This method, termed EXP‐J, exhibited remarkable sensitivity and specificity, demonstrating the promise of Cas12j as a potent tool for miRNA detection. The EXP‐J method was further applied to detect miRNAs in plasma samples from lung cancer patients. The detection results show a strong correlation with standard reverse transcription quantitative polymerase chain reaction (RT‐qPCR) assays, underscoring the reliability and clinical relevance of the Cas12j‐based detection system. The present study is expected to not only elucidate the unique trans‐cleavage dynamics of Cas12j, but also pave the way for exploring Cas12j applications beyond miRNA detection to a broader spectrum of molecular diagnostics.

## 2 Results

### 2.1 Exploration of Optimal Buffer Conditions for Enhancing Cas12j Trans‐Cleavage Efficiency

**Figure 1:**
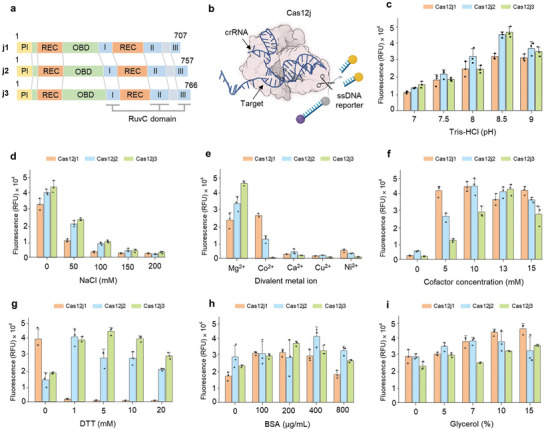
Exploration of optimal buffer conditions for enhancing Cas12j trans‐cleavage efficiency. a) Schematic representation of three Cas12j variants showing their domain organization. Each variant contains four domains: PI domain in yellow, oligonucleotide binding domain (OBD) in green, nucleic acid recognition domain (REC) in orange, and DNase domain (RuvC) in blue. b) Illustration of the Cas12j trans‐cleavage fluorescence assay using ssDNA target and ssDNA reporter with fluorophore and quencher. Trans‐cleavage activity evaluation of three Cas12j variants (Cas12j1 in orange, Cas12j2 in blue, and Cas12j3 in green) under different buffer conditions. Factors evaluated include c) pH, d) salt concentration, e) specific metal ion, f) optimal metal ion concentration, g) DTT concentration, h) BSA concentration, and i) glycerol concentration. The mean fluorescence intensities from three technical replicates are represented as bars and individual dots indicate raw fluorescence data from each experiment. Error bars indicate standard deviations (SD).

In this study, three different variants of the Cas12j protein family were investigated for their trans‐cleavage ability. These variants are unified by structural features, including an N‐terminal PAM interaction (PI) domain and a unique C‐terminal RuvC nuclease domain, coupled with sequence homologies ranging from 38% to 45% (**Figure** **1**a).^[^ ^8^, ^9^ ^]^ The Cas12j proteins were efficiently overexpressed in *Escherichia coli* and isolated without the need for solubility‐enhancing tags (Table S1, Supporting Information). The Cas12j family was subjected to a carefully optimized two‐step purification protocol, achieving purity levels of nearly 95% (Figure S1, Supporting Information). Remarkably, the yield per liter was exceptionally high: Cas12j1 at ≈15 mg L^−1^, Cas12j2 at ≈25 mg L^−1^, and Cas12j3 at ≈30 mg L^−1^. This represents a significant improvement over the yields of the Cas12a and Cas13a protein families, which stand at merely ≈5 mg L^−1^ and 0.5 mg L^−1^, respectively.^[^ ^5^, ^11^ ^]^ To address the inherent challenges of low solubility and purification yields typical of Cas proteins, a strategic intervention was made by incorporating over 2 M salt concentration in the purification buffer. This adjustment significantly increased the solubility of the Cas12j proteins, thereby facilitating the maximization of recovery of the proteins.

To measure the trans‐cleavage activity of Cas12j protein family, a reporter molecule containing a fluorescent dye, a quencher, and a 5′‐TTATT‐3′ sequence was used. Initiation of trans‐cleavage was driven by a 24 nucleotides (nt) single‐stranded DNA (ssDNA) designed to be complementary to the crRNA sequence (Figure 1b). The oligonucleotides used in the experiments are listed in Tables S2–S4 (Supporting Information). The trans‐cleavage activity of Cas proteins is dependent on parameters that affect the stability of the protein and the dynamic interplay between crRNAs and target DNA.^[^ ^12^ ^]^ Therefore, the trans‐cleavage efficiency of Type V effector proteins can be influenced by buffering factors. To investigate the optimal buffer composition conducive to the trans‐cleavage activity of the Cas12j variants, a comprehensive study of changes in pH, salt concentration, and cofactor was performed. A peak of trans‐cleavage activity was observed at pH 8.5 when pH was adjusted between 7 and 9 at 0.5 intervals (Figure 1c; Figure S2a, Supporting Information). Furthermore, optimal activity was reached in the absence of salt when the NaCl concentration was varied from 0 to 200 mM (Figure 1d; Figure S2b, Supporting Information). Regarding cofactor preferences, Cas12j2 and Cas12j3 showed a discernible affinity for Mg^2+^, whereas Cas12j1 showed a preference for Mg^2+^ and Co^2+^ (Figure 1e; Figure S2c, Supporting Information). To further investigate the effects of divalent metal ion concentrations on the Cas12j variants, each was tested with its preferred ion: Co^2^⁺ for Cas12j1 and Mg^2^⁺ for Cas12j2 and Cas12j3. In the presence of target ssDNA, the peak activities for Cas12j1, Cas12j2, and Cas 12j3 were reached at 10 mM Co^2^⁺, 10 mM Mg^2^⁺, and 13 mM Mg^2^⁺, respectively (Figure 1f; Figure S2d, Supporting Information). This extensive analysis elucidates the optimal buffer conditions for the trans‐cleavage activity of Cas12j variants and advances the understanding of the nuanced interplay between buffer composition and enzyme efficiency, paving the way for optimized applications in molecular diagnostics.

To enhance the trans‐cleavage efficiency of Cas12j, the effects of dithiothreitol (DTT), bovine serum albumin (BSA), and glycerol in the reaction buffer were also investigated. DTT is essential for maintaining the redox state and integrity of the disulfide bonds within the Cas proteins, which are critical for maintaining their inherent nucleic acid binding properties.^[^ ^13^ ^]^ BSA plays a bifunctional role, providing stability and preventing protein adhesion to the reaction vessel, thereby ensuring maximum enzyme availability for the reaction.^[^ ^14^ ^]^ Glycerol plays a key role in maintaining the 3D conformation of Cas proteins, facilitating interactions between the Cas/crRNA complex and the target DNA.^[^ ^15^ ^]^ A systematic approach was used to determine the optimal concentration of these additives in the reaction buffer. Concentrations of DTT were varied from 0 to 20 mM, BSA from 0 to 800 µg mL^−1^, and glycerol from 0% to 20%, and the trans‐cleavage activities of the Cas12j variants were evaluated under each condition. The results described a significant increase in fluorescence with the inclusion of DTT, with the trans‐cleavage activity of Cas12j2 peaking at 1 mM and Cas12j3 at 5 mM, indicating an optimal redox environment for these variants (Figure 1g; Figure S3a, Supporting Information). In contrast, a significant decrease in the activity of Cas12j1 was observed upon addition of DTT, suggesting a distinct redox sensitivity of this variant. In addition, incorporation of BSA into the buffer improved the trans‐cleavage activity of all three Cas12j variants. Optimal performance was observed for Cas12j1 and Cas12j3 at a BSA concentration of 200 µg mL^−1^, while Cas12j2 showed superior activity at 400 µg mL^−1^ (Figure 1h; Figure S3b, Supporting Information). This highlights the stabilizing and anti‐adhesive role of BSA, which meets the different requirements of each Cas12j variant. Glycerol concentration showed a variant‐specific effect on trans‐cleavage activity. High fluorescence was observed at a glycerol concentration of 15% for Cas12j1, 7% for Cas12j2, and 20% for Cas12j3 (Figure 1i; Figure S3c, Supporting Information). This variability indicates the individual structural and functional intricacies of each variant in response to glycerol. By judiciously incorporating these additives, we have delineated the optimal buffer conditions conducive to the trans‐cleavage efficiency of each Cas12j variant. This optimization suggests the way for exploiting the trans‐cleavage potential of Cas12j in nucleic acid detection, thereby increasing the reliability and efficacy of Cas12j‐based diagnostic systems.

### 2.2 Evaluation of Optimal Reaction Conditions for Enhancing Cas12j Trans‐Cleavage Efficiency

**Figure 2:**
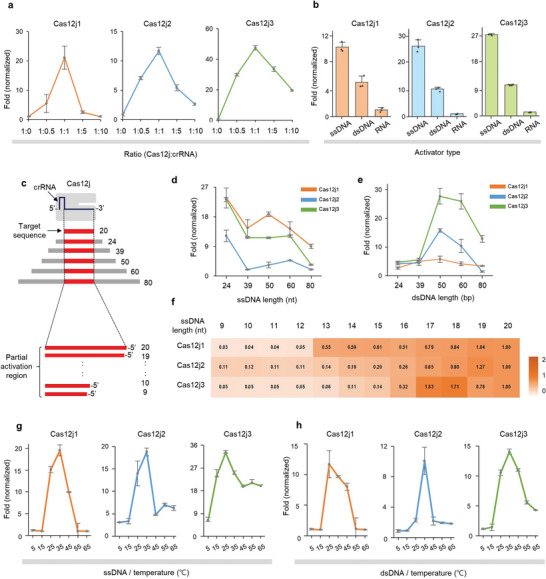
Evaluation of optimal reaction conditions for enhancing Cas12j trans‐cleavage efficiency. a) Evaluation of trans‐cleavage activity by different concentrations of crRNA. Optimal stoichiometry was determined a 1:1 ratio of Cas12j to crRNA. b) Evaluation of trans‐cleavage activity by different target nucleic acid types. Activity levels were assessed under identical conditions using equal amounts of target ssDNA, dsDNA, and RNA. c) Schematic representation of Cas12j/crRNA with target DNA of different lengths. The region containing the target sequence is highlighted in red. The extended length beyond 20 nt is added to both the 5′ and 3′ ends. The length of target used in the reaction is shown to the right of the target DNA. Target nucleic acids less than 20 nt in length were excised from the 3′ end of the crRNA. Evaluation of trans‐cleavage activity by different target length. Assays were performed with target d) ssDNA and e) dsDNA spanning lengths from 24 to 80 nt. f) Heatmap showing the relative trans‐cleavage activity by different target lengths, with the 20 nt target serving as the reference. Assays were performed with truncated ssDNA sequences from 9 to 20 nt. The mean fluorescence intensities from three technical replicates were used. Temperature dependence of Cas12j trans‐cleavage activity. Assays were performed with target g) ssDNA or h) dsDNA at different temperature. Fold (normalized) indicates relative changes by dividing the fluorescence in the presence of a target by the fluorescence in its absence. Error bars indicate SD of three replicates. Individual dots in bar graph indicate raw data from each experiment.

After carefully optimizing buffers tailored to the three Cas12j variants, we set out to elucidate the reaction parameters that significantly influence their trans‐cleavage efficiency. In particular, we focused on the concentration of crRNA, the type and length of target nucleic acids, and the reaction temperature, all of which are considered critical for trans‐cleavage activity. **Figure** **2**a and Figure S4a (Supporting Information) show a pronounced effect of crRNA concentration on the trans‐cleavage efficiency of the Cas12j family. An optimal stoichiometry for maximizing trans‐cleavage activity was found to be characterized by a 1:1 ratio of protein to crRNA. Exceeding the equilibrium concentration of crRNA results in a decrease in Cas12j activity. This plateau contrasts with typical Cas12a dynamics, where trans‐cleavage activity increases as the molar concentration of crRNA surpasses that of Cas12a protein.^[^ ^16^ ^]^ This observation highlights the inherent variability in crRNA dependence between different CRISPR systems.

The nature of the target nucleic acids is another critical parameter for the trans‐cleavage activity of Cas12j variants (Figure 2b; Figure S4b, Supporting Information). It was found that both ssDNA and double‐stranded DNA (dsDNA) targets can act as inducers of trans‐cleavage, whereas RNA target was unsuitable for the initiation of the trans‐cleavage cascade. A notable observation was the superior inducibility of ssDNA targets, which increased trans‐cleavage activity by 3‐ to 4‐fold compared to dsDNA of identical sequence and length. This difference in activity underscores the intrinsic affinity and efficiency of the Cas12j variants for ssDNA substrates. In an effort to explore the effect of overhang and target sequence length, on the trans‐cleavage efficiency of Cas12j, a structured investigation was performed (Figure 2c). First, both ssDNA and dsDNA were used as target substrates. The length of target substrates varied from 24 to 80 nt, including the target recognition site of 20 nt. As dsDNA targets require an additional 4 bp apart from the 20 bp complementary to the crRNA due to the need for a PAM sequence, we chose target lengths starting from 24 nt for both experiments. With the ssDNA activators, all Cas12j variants provided a pronounced peak of trans‐cleavage activation at a length of 24 nt (Figure 2d; Figure S4c, Supporting Information). This activity exhibited different patterns between 39 nt and 60 nt depending on the variant, but overall, the activity sharply declined at a length of 80 nt. In contrast, with dsDNA activators, the peak of trans‐cleavage efficiency was observed at a length of 50 base pairs (bp) with a trend of decreasing efficiency for both shorter and longer sequences (Figure 2e; Figure S4d, Supporting Information). A subsequent investigation was conducted to determine the minimum partial target length to activate the trans‐cleavage of Cas12j variants (Figure 2f; Figure S5a,b, Supporting Information). In this experiment, ssDNA target activators ranging in length from 9 to 20 nt were used, and the trans‐cleavage activity for each target was compared to a 20 nt target. The results revealed that sequences truncated from the 5′ end retained trans‐cleavage activity, while those truncated from the 3′ end, near the PAM motif, did not. The heatmap results indicate that all variants require a complementary sequence of at least 17 nt from the 3′ end to significantly enhance their trans‐cleavage activity.

To further elucidate the optimal temperature conducive to the trans‐cleavage activity of the Cas12j variants, a temperature‐centric assay was performed from 5 to 65 °C. When ssDNA activator was used, both Cas12j1 and 2 showed a peak activity at 35 °C, whereas Cas12j3 showed optimal activity at 25 °C (Figure 2g; Figure S5c, Supporting Information). On the other hand, with dsDNA as activator, Cas12j1 reached its peak activity at 25 °C, while both Cas12j2 and Cas12j3 showed peak activities at 35 °C (Figure 2h; Figure S5d, Supporting Information). These analyses provide a deeper understanding of the operational parameters that govern the trans‐cleavage efficiency of Cas12j variants.

### 2.3 Effect of Target DNA Mismatch on Cas12j Trans‐Cleavage Efficiency

**Figure 3:**
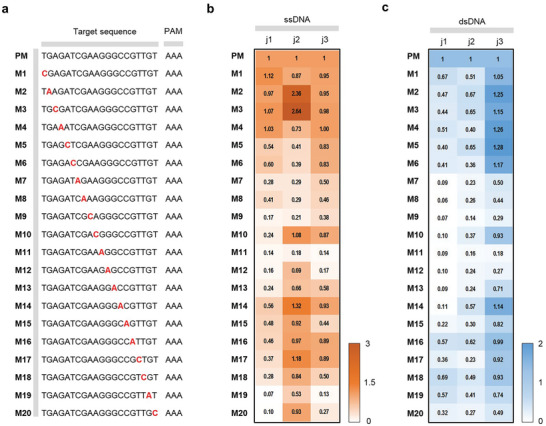
Effect of target DNA mismatch on Cas12j trans‐cleavage efficiency. a) Depicted sequences show PM and mismatches marked with “M” and mismatch number. The mismatched nucleotide is highlighted in bold red. Heatmap showing Cas12j variant activity onto mismatched b) ssDNA and c) dsDNA target, with the PM serving as the reference. The mean fluorescence intensities from three technical replicates were used.

The precise elucidation of the target recognition specificity of the Cas/crRNA complex is crucial for the advancement of diagnostic applications. To investigate the sequence specificity of Cas12j‐mediated trans‐cleavage reactions, we designed an experimental setup in which single nucleotide mutations were introduced into 20 different positions of target sequences (**Figure** **3**a). The analysis was performed using ssDNA or dsDNA as target activators. Variations in trans‐cleavage activity are referenced against the perfect match (PM) sequence. When ssDNA was used as activator, Cas12j1 showed a decrease in trans‐cleavage activity except for positions M1‐M4 distal to the PAM site (Figure 3b; Figure S6, Supporting Information). In addition, the pronounced decrease in activity at positions M9‐M13, M19, and M20 represents a high sequence specificity of Cas12j1. Meanwhile, Cas12j2 showed a significant decrease in activity, especially at positions M5‐M9 and M11, highlighting their key roles in sequence recognition. Cas12j3, in contrast to the other variants, showed attenuated trans‐cleavage activity at positions M7‐M9 and M11‐M13, while positions M19 and M20, close to the PAM site, showed almost complete elimination of activity. When dsDNA was used as an activator, decreased activity at positions M19 and M20, proximal to the PAM site, was observed in all Cas12j variants (Figure 3c; Figure S7, Supporting Information). Moreover, Cas12j1 showed an increased susceptibility to point mutations over the entire spectrum examined, with a strong loss of trans‐cleavage activity at positions M7‐M15. Cas12j2 also showed a consistent decrease in trans‐cleavage activity in response to point mutations at all positions, with a more pronounced decrease at positions M7‐M13. Cas12j3 showed a more variable sequence specificity; mutations at M7‐M9, M11, and M12 culminated in decreased activity, whereas mutations at positions M1‐M6 and M14 had a negligible effect on trans‐cleavage efficacy. The data obtained in this study provide an understanding of the sequence specificity conferred by Cas12j variants in trans‐cleavage reactions. This not only highlights the potential for fine‐tuning Cas12j variants for enhanced specificity, but also provides a foundation for the development of diagnostic applications that take advantage of the molecular precision inherent in the Cas12j system.

### 2.4 Application of Cas12j to CRISPR Diagnostics: EXP‐J Reaction for miRNA Detection

After unveiling the detailed characteristics of Cas12j variants, we aimed to leverage these findings for molecular diagnostics. MiRNAs, known for their central role in post‐transcriptional gene regulation, are often dysregulated in a variety of pathological conditions, including cancer, cardiovascular disease, and neurological disorders.^[^ ^10^, ^17^ ^]^ This makes their expression profiles invaluable as biomarkers that provide insight into disease onset, progression and response to therapy.^[^ ^18^ ^]^ Consequently, the development of advanced miRNA detection methods that exploit the properties of Cas12j variants could contribute to the early and accurate diagnosis of disease and improve the understanding of intricate molecular mechanisms underlying various health conditions.

**Figure 4:**
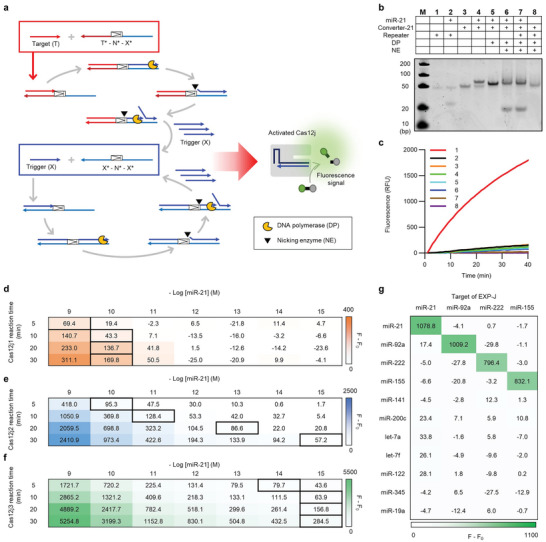
EXP‐J reaction for miRNA detection. a) Schematic representation of EXP‐J reaction for miRNA detection. The target (T) hybridizes to the converter (T* – N* – X*), allowing elongation by DP and subsequent formation of a nicking site. This site is cleaved by an NE, producing a trigger (X). The trigger is then amplified by the repeater (X* – N* – X*), which activates Cas12j‐mediated trans‐cleavage of a reporter DNA, culminating in a fluorescence signal indicating the presence of target miRNA. b) PAGE analysis of EXP‐J reaction (M: ultra‐low range DNA ladder, 1: repeater, 2: repeater + miR‐21, 3: converter‐21, 4: converter‐21 + miR‐21, 5: converter‐21 + miR‐21 + DP, 6: converter‐21 + miR‐21 + DP + NE, 7: converter‐21 + repeater + miR‐21 + DP + NE, 8: converter‐21 + repeater + DP + NE). The experiments were independently replicated three times. The final concentrations of converter‐21, repeater, miR‐21, DP, NE are 50 nM, 50 nM, 50 nM, 0.1 and 0.25 U µL^−1^, respectively. The reaction was conducted at 55 °C for 10 min. **c** Fluorescence validation of EXP‐J reaction components (1: miR‐21 + converter‐21 + repeater + DP + NE + Cas12j2/crRNA + reporter DNA, 2: converter‐21 + repeater + DP + NE + Cas12j2/crRNA + reporter DNA, 3: miR‐21 + repeater + DP + NE + Cas12j2/crRNA + reporter DNA, 4: miR‐21 + converter‐21 + DP + NE + Cas12j2/crRNA + reporter DNA, 5: miR‐21 + converter‐21 + repeater + DP + Cas12j2/crRNA + reporter DNA, 6: miR‐21 + converter‐21 + repeater + NE + Cas12j2/crRNA + reporter DNA, 7: miR‐21 + converter‐21 + repeater + DP + NE + reporter DNA, 8: miR‐21 + converter‐21 + repeater + DP + NE + Cas12j2/crRNA). The experiments were independently replicated three times. The final concentrations of converter‐21, repeater, miR‐21, DP, NE, Cas12j2/crRNA, and reporter DNA are 5 nM, 10 nM, 100 pM, 0.1 and 0.25 U µL^−1^, 400, and 400 nM, respectively. Comparison of EXP‐J reaction efficiency using d) Cas12j1, e) Cas12j2, and f) Cas12j3, respectively. EXP‐J reactions were performed with different Cas12j reaction times and miR‐21 concentrations. Thick bordered cells represent the detection limit above the threshold value (F – F_0_ + 3SD of blank samples). F and F_0_ represent the fluorescence intensities after EXP‐J reactions with and without miR‐21 from three technical replicates. The final concentrations of converter‐21, repeater, DP, NE, Cas12j/crRNA, and reporter DNA are 5 nM, 10 nM, 0.1 and 0.25 U µL^−1^, 100, and 400 nM, respectively. g Selectivity and versatility of the EXP‐J reaction. The heatmap shows F – F_0_ values of each EXP‐J reaction targeting four different miRNAs (miR‐21, miR‐92a, miR‐222, and miR‐155) in the presence of various miRNAs. Strong fluorescence signal was observed only when the corresponding miRNA to the target of EXP‐J reaction was present. F and F_0_ represent the fluorescence intensities after EXP‐J reactions with and without miRNAs from three technical replicates. The final concentrations of converters, repeater, miRNAs, DP, NE, Cas12j3/crRNA, and reporter DNA are 5 nM, 10 nM, 100 pM, 0.1 and 0.25 U µL^−1^, 100, and 400 nM, respectively.

We designed the EXP‐J reaction for miRNA detection based on the revealed characteristics of Cas12j variants, including the strong trans‐cleavage activity toward short ssDNA (**Figure** **4**a). The structural design of the EXP‐J reaction system includes two coupled amplification templates: the converter (T* – N* – X*) and the repeater (X* – N* – X*). Upon introduction of the target miRNA (T), it binds to the converter, which is then elongated by the DNA polymerase (DP), resulting in the formation of a double‐stranded nicking sequence that serves as a substrate for the nicking enzyme (NE). The DP and NE then work synergistically to produce the trigger (X). The trigger is further amplified by the repeater, which prolifically generates the X sequence through an exponential cascade reaction. Finally, the generated triggers activate the trans‐cleavage of Cas12j to cleave adjacent reporter DNA. This allows the emission of a strong fluorescence signal indicating the presence of the target miRNA. The designed EXP‐J reaction can expand the range of target miRNAs by simply changing the T* sequence of the converter. Even when targeting different miRNAs, the Cas12j/crRNA remains unchanged in the different EXP‐J reactions because the same trigger is generated regardless of the target miRNA sequence. This ensures the stability and versatility of the EXP‐J reaction, suggesting its potential as a reliable and adaptable miRNA detection platform. In addition, the Cas12j/crRNA can alleviate false‐positive signals of EXPAR reaction, by double‐checking the amplified products.^[^ ^19^ ^]^ Therefore, the EXP‐J reaction can balance the high amplification efficiency but accompanying false positive signals of the EXPAR reaction with the high specificity of Cas12j.

To test the feasibility of the EXP‐J reaction, miR‐21 as model target (Table S5, Supporting Information). We first performed polyacrylamide gel electrophoresis (PAGE) analysis to confirm the reaction (Figure 4b). Notably, lanes 3 and 4 clearly show the specific binding between miR‐21 and converter‐21, in contrast to the lack of interaction between miR‐21 and the repeater as observed in lanes 1 and 2. The introduction of DP elongated miR‐21 along converter‐21, resulting in a prominent band and a noticeable shift in its position (lane 5 in Figure 4b). The subsequent addition of NE resulted in the appearance of trigger band located at the bottom of the gel (lane 6 in Figure 4b), which was far intensified by the inclusion of the repeater (lane 7 in Figure 4b). In the absence of the target, no trigger or by‐product bands were observed (lane 8 in Figure 4b). This result clearly confirms that the designed EXP‐J system works only in the presence of the target miRNA. The EXP‐J reaction was further confirmed by fluorescence measurements under different reaction components. Significant fluorescence enhancement was observed only when both the target and all EXP‐J reaction components were present (curve 1 in Figure 4c). No fluorescence signal enhancement was observed when one of the reaction components was missing, despite the presence of the target (curves 3–8 in Figure 4c). Even with a complete set of EXP‐J reaction components, fluorescence signal enhancement was not observable in the absence of the miR‐21 target (curve 2 in Figure 4c). These results clearly demonstrated that the initiation and successful completion of the EXP‐J reaction were highly dependent on the presence of the target miRNA and the collaborative functioning of all reaction components. After confirming feasibility, the EXP‐J reaction conditions, including reaction temperature, trigger length, and concentrations of converter, repeater, rNEbuffer 3.1, and Cas12j/crRNA, were optimized. As a result, the following optimal conditions were determined: 55 °C for reaction temperature, 19 nt trigger length, 5 nM converter, 10 nM repeater, 0.2× rNEbuffer 3.1, and 100 nM Cas12j/crRNA (Figures S8–S13, Supporting Information). These EXP‐J conditions were used for the subsequent experimental procedures.

In the following analysis, we attempted to determine the detection efficiency of the three Cas12j variants in the EXP‐J reactions. In particular, the EXP‐J reactions were performed over different Cas12j reaction times of 5, 10, 20, 30 min and miR‐21 concentrations ranging from 1 fM to 1 nM to elucidate the kinetics and sensitivity of miRNA detection (Figure 4d–f; Figure S14, Supporting Information). The detection limit was estimated as the lowest miR‐21 concentration exceeding the threshold value (F – F_0_ + 3SD of blank samples) and marked by a thick border for each reaction time of the Cas12j variants. F and F_0_ represent the fluorescence intensities after EXP‐J reactions with and without target miRNA. We compared the F – F₀ values to accurately represent the fluorescence increase solely attributable to the target. Interestingly, the experimental results revealed different performance characteristics among the Cas12j variants. First, Cas12j1 showed poor efficiency with a marginal increase in fluorescence even after 30 min of Cas12j1 reaction time, detecting miR‐21 down to only 10 pM (Figure 4d). This lower detection efficiency was attributed to the presence of DTT in the EXP‐J reaction buffer, which suppressed the trans‐cleavage activity of Cas12j1 as shown in Figure 1g. Due to this inhibitory effect, Cas12j1 was considered unsuitable for the EXP‐J reaction and was excluded from subsequent experiments. For Cas12j2, examination over a time gradient revealed a peak in detection efficiency at a 30 min reaction, detecting miR‐21 at a low concentration of 1 fM (Figure 4e). Reducing the reaction time of Cas12j2 to 20, 10, and 5 min resulted in lower detection limits of 100 fM, 10 pM, and 100 pM due to the reduced signal intensity. Significantly, Cas12j3 showed the fastest fluorescence increase, enabling the detection of miR‐21 down to 1 fM within 10 min reaction time (Figure 4f). Based on this result, we selected the 10 min reaction time of Cas12j3 for the final EXP‐J reaction. This condition was employed in subsequent experiments. Compared to previously reported CRISPR diagnostics for miRNA, the EXP‐J reaction provides comparable or lower detection limit within a shorter reaction time (Table S6, Supporting Information). This excellent performance was attributed to the precise investigation of Cas12j variants and their appropriate incorporation into the EXP‐J reaction. The differential kinetic and sensitivity profiles of the Cas12j variants, as unraveled in this investigation, not only provide a sophisticated understanding of their operational dynamics, but also lay the groundwork for their use to improve diagnostic accuracy and rapidity.

As mentioned above, the EXP‐J reaction can expand the target miRNAs by simply changing the converter. Therefore, we developed additional EXP‐J reactions for three different miRNAs (miR‐92a, miR‐222, and miR‐155). The EXP‐J reactions for three miRNAs showed similar detection efficiencies to miR‐21 (Figure 4g). Furthermore, when four types of EXP‐J reactions were performed with various miRNAs (miR‐21, miR‐92a, miR‐222, miR‐155, miR‐141, miR‐200c, let‐7a, let‐7f, miR‐122, miR‐345, and miR‐19a), an increase in fluorescence signal was observed only when the corresponding target miRNA was present, highlighting the high selectivity and universality of the EXP‐J reaction (Figure S15, Supporting Information). We anticipate that the EXP‐J reaction opens a compelling avenue for miRNA detection, underscored by precision and enhanced signal output.

### 2.5 Application of EXP‐J Reaction to Lung Cancer Diagnosis

**Figure 5:**
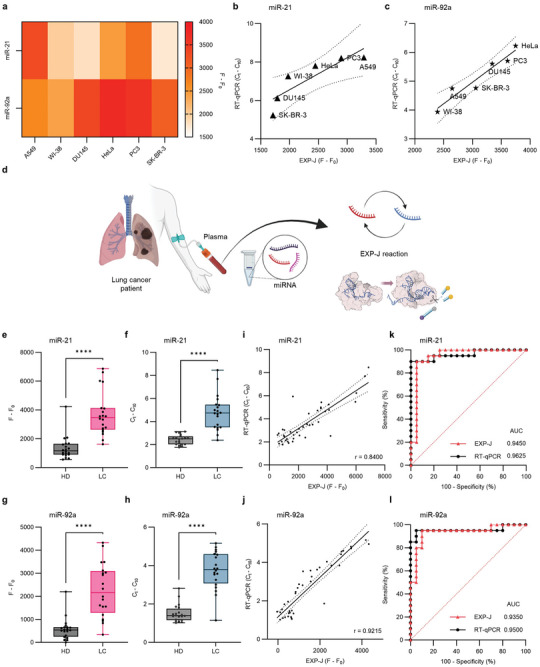
EXP‐J reaction for lung cancer diagnosis. a) Heatmap showing the expression patterns of miR‐21 and miR‐92a in six cell lines (A549, WI‐38, DU145, HeLa, PC3, and SK‐BR‐3) using the EXP‐J assays. Each assay was repeated three times. Analytical agreement between EXP‐J assay and RT‐qPCR for b) miR‐21 and c) miR‐92a across cell lines. Data points represent the mean of three replicates of each measurement for each method. Linear fitting of data points was performed to generate linear correlation curves. Dashed lines represent 95% confidence bands of the best fit line. d) Schematic representation of EXP‐J reaction for lung cancer diagnosis (Created by Biorender.com). EXP‐J reaction was performed to detect miR‐21 and miR‐92a from the clinical plasma samples. Analysis of e,f) miR‐21 and g,h) miR‐92a from lung cancer patients (n = 20) and healthy controls (n = 20) by e,g) EXP‐J assay and f,h) RT‐qPCR. Both methods show the overexpression of miR‐21 and miR‐92a in plasma samples from lung cancer patients. Data points represent the mean of three replicates of each measurement for each method. Error bars indicate SD. Statistical analysis was performed using a two‐tailed t‐test with Welch correction where the asterisks (****p ≤ 0.0001) denote significant differences. All statistical analyses were performed at 95% confidence level. Correlation between EXP‐J assay and RT‐qPCR results for i) miR‐21 and j) miR‐92a detection. Data points represent the mean of three replicates of each measurement for each method. Linear fitting of data points was performed to generate linear correlation curves. Dashed lines represent 95% confidence bands of the best fit line. ROC curves for differentiation of lung cancer from healthy controls by k) miR‐21 and l) miR‐92a detection results using EXP‐J assay and RT‐qPCR. The AUC for each of the ROC curves is annotated.

Lung cancer is one of the leading causes of cancer‐related deaths worldwide and often presents few or no symptoms in its early stages, making early detection challenging. In addition, the difficulty in obtaining lung tissue samples makes liquid biopsy essential.^[^ ^20^ ^]^ Since miR‐21 and miR‐92a have been shown to be involved in the pathogenesis of cancer,^[^ ^21^ ^]^ the EXP‐J reaction was applied to detect miR‐21 and miR‐92a. First, six different cell lines, including A549 (lung cancer), WI‐38 (lung epithelial), DU145 (prostate cancer), HeLa (cervical cancer), PC3 (prostate cancer), and SK‐BR‐3 (breast cancer), were examined by the EXP‐J assay. **Figure** **5**a and Figure S16 (Supporting Information) show the expression patterns for miR‐21 and miR‐92a from the tested cell lines, which are in good agreement with previous reports.^[^ ^21^, ^22^ ^]^ Moreover, the analytical agreement between the EXP‐J assay and conventional RT‐qPCR (Table S7, Supporting Information) was examined and Pearson correlation coefficients of 0.8738 and 0.9618 were obtained (Figure 5b,c). This indicates a robust analytical capability of the EXP‐J reactions. Therefore, we expect that the proposed strategy can be used to reliably determine miRNAs in complex heterogeneous biological samples.

Next, we applied the EXP‐J reactions to determine the expression levels of miR‐21 and miR‐92a in plasma samples from two cohorts: lung cancer patients (n = 20, Table S8, Supporting Information) and healthy controls (n = 20, Table S9, Supporting Information). Total miRNA was extracted from the clinical plasma samples and the EXP‐J reaction was performed for miR‐21 and miR‐92a, respectively (Figure 5d). The data obtained from the EXP‐J assay revealed a significant overexpression of miR‐21 and miR‐92a in the plasma of lung cancer patients (Figure 5e,g; Figure S17, Supporting Information), which was also confirmed by RT‐qPCR results (Figure 5f,j). When the analytical agreement between the two methods was examined, Pearson correlation coefficients of 0.8400 and 0.9215 were obtained (Figure 5i,j). Receiver operating characteristic (ROC) curve analysis for the EXP‐J assay demonstrated that miR‐21 and miR‐92a effectively differentiated lung cancer patients from healthy individuals with area under the curve (AUC) values of 0.9450 (95% CI: 0.8640‐1.000) and 0.9350 (95% CI: 0.8523‐1.000), respectively (dotted red curves in Figure 5k,l). The optimal cut‐off value was 2209 for miR‐21, resulting in 90% sensitivity and 95% specificity, and the value was 1182 for miR‐92a, resulting in 80% sensitivity and 95% specificity. The AUC values of RT‐qPCR were comparable to those of the EXP‐J assay (dotted black curves in Figure 5k,l). This substantial correlation of AUC values underscores the diagnostic efficacy of the EXP‐J assay in liquid biopsy. The successful application of the EXP‐J assay in authentic clinical samples opens a promising avenue for its future use as a reliable diagnostic platform. We emphasize that the unveiling of Cas12j trans‐cleavage activity served as a critical enabler for the development of the EXP‐J assay and thus contributed to the advancement of CRISPR diagnostics.

## 3 Discussion

The Cas12j family, which is characterized by its smaller size compared to conventional Cas12 proteins, has emerged as a promising candidate for gene editing and diagnostic applications. Recently, the gene editing capabilities of Cas12j2 and Cas12j8 have been verified,^[^ ^7^, ^23^ ^]^ but the application of Cas12j family in diagnostics has not yet been reported. The trans‐cleavage activity of Cas12 families serves as a fundamental mechanism in CRISPR diagnostics to increase sensitivity and selectivity. To maximize the trans‐cleavage activity of Cas12j and thus apply it to CRISPR diagnostics, we meticulously investigated the optimal buffer and reaction conditions. In particular, we focused on Cas12j1, 2, and 3 variants because they were evidenced at the protein level. The carefully evaluated results not only provide valuable insights into the operational nuances of Cas12j, but also confirm its suitability for nucleic acid detection, contributing to broaden the landscape of CRISPR‐based diagnostics. The comparison of Cas proteins for diagnostic applications is provided in Table S10 (Supporting Information). In general, Cas12‐based diagnostics are more stable than Cas13‐based diagnostics, given that RNA is more prone to degradation by nucleases.^[^ ^24^ ^]^ Additionally, most isothermal amplification methods produce DNA amplicons regardless of target type (RNA or DNA), making Cas12‐based diagnostics more versatile.^[^ ^25^ ^]^ Cas12j offers an advantage in diagnostic applications due to its smaller size (80‐89 kDa) and shorter crRNA sequences (44 nt) compared to those of Cas9 (160‐170 kDa, 100 nt), Cas12a (130‐150 kDa, 40–44 nt), Cas13a (137‐150 kDa, 62 nt), and Cas12f1 (50‐70 kDa, 140 nt). This compact design has the potential to facilitate system development and reduce costs. Despite the smaller size of Cas12f1 than Cas12j, its longer crRNA (≈4‐fold longer than Cas12j) makes it less favorable in diagnostics. Furthermore, the yield of purified protein for Cas12j is 5‐ to 25‐fold higher than for other Cas proteins. The high productivity of Cas12j can be attributed to its small size and the rigid activation of its trans‐cleavage activity, which targets short ssDNA that are scarce in microbial systems. This distinct property of Cas12j results in minimal harm to microbial survival, thereby enhancing microbial expression.

A notable discovery of the current study is the trans‐cleavage activation of Cas12j with short target nucleic acids, a unique characteristic among Cas12 proteins. This property likely relates to the distinct structure of its REC II domain,^[^ ^8^, ^9^ ^]^ particularly a flexible loop spanning amino acids 511–535 (PDB ID: 7YLT). As shown in Figure S18 (Supporting Information), this loop is crucial for guiding the target strand to the active site. Previous studies have shown that target sequence recognition leads to conformational changes in the RuvC and REC II domains, activating the DNase function of Cas12j.^[^ ^8^ ^]^ The specialized domain structure and narrower target binding pocket compared to Cas12a may influence enzymatic activity based on target strand length. We found that 17 nt target sequences are necessary to activate Cas12j (Figure 2f). This indicates that a target sequence length of 17 nt is sufficient to induce the conformational changes for DNase activity of Cas12j. However, ssDNA targets longer than 24 nt impair its non‐specific nuclease function, likely due to steric hindrance. This hindrance obstructs non‐specific ssDNA entry into the catalytic site, reducing cleavage efficiency. Additionally, our analysis of 20 nt target sequence variations revealed minimal sequence specificity at the 5′ end (M1‐M4 site, Figure 3), suggesting these sequences play a minor role in structural dynamics. These findings highlight the delicate balance in target length and sequence composition required for optimal trans‐cleavage activity of Cas12j.

After unveiling the characteristics of Cas12j, we designed the EXP‐J reaction, which generates short ssDNA for Cas12j activation. The EXP‐J reaction integrates the amplification power of EXPAR with the trans‐cleavage activity of Cas12j, enabling robust and sensitive miRNA detection. The EXP‐J assay achieves a detection limit of 1 fM with a rapid reaction time of 40 min. For comparison, we introduced Cas12a, which is widely used in CRISPR‐based diagnostics, into the EXP‐J system. As shown in Figure S19 (Supporting Information), Cas12a exhibited similar detection sensitivity to Cas12j3. However, Cas12a showed higher background noise (represented as a red dotted line) compared to Cas12j (represented as a blue dotted line), due to its lower specificity and higher trans‐cleavage activity. This finding suggests that Cas12j3 is a better fit within the context of EXP‐J assay.

Notably, lung cancer patients were successfully discriminated from healthy donors based on the upregulation of miR‐21 and miR‐92a measured by the EXP‐J assay. The importance of these results is underscored by the advantages of liquid biopsy as a rapid, minimally invasive, and cost‐effective method for disease diagnosis and prognosis.^[^ ^26^ ^]^ In particular, the miRNA profile from blood samples stands out for its potential in facilitating early disease detection, improved diagnostic accuracy, and personalized therapeutic intervention.^[^ ^10^, ^17^ ^]^ Considering these factors, the successful application of the EXP‐J assay in plasma samples suggests the feasibility of its advancement as a reliable diagnostic platform in the clinical landscape.

Although we unveiled the trans‐cleavage activity of Cas12j variants and provided a foundation for the development of Cas12j‐based diagnostic platforms, there is still room for improvement. First, engineering Cas12j variants for enhanced specificity and reduced off‐target effects is an imperative project that will significantly advance CRISPR diagnostics. Moving forward, we aim to develop improved Cas12j variants with distinct cleavage capabilities by leveraging the properties and insights for Cas12j gained from the current study. Second, despite the promising aspects of miRNA profiling in lung cancer diagnosis, challenges such as inter‐individual variability in miRNA expression, tumor heterogeneity, and the presence of confounding factors should be considered. Furthermore, as miRNA‐based diagnostics are still in the developmental stage, the establishment of standardized protocols and reference ranges is needed to ensure the reproducibility and clinical utility of these tests. In this regard, expanding the range of detectable nucleic acids would be beneficial to broaden the applicability of the present assay to different types of cancer and other diseases.

In conclusion, this study sheds light on the previously obscured trans‐cleavage activity of Cas12j variants and establishes their promising role in CRISPR diagnostics. Through careful investigation, we have characterized the target preferences and mismatch specificities of these variants and optimized the reaction conditions for their use. The development of the EXP‐J assay, which utilizes the robust trans‐cleavage activity of Cas12j on short ssDNA, marks a significant advancement in miRNA detection. The translational applicability of the assay is proved by its successful use in the detection of miRNAs in plasma samples from lung cancer patients. Overall, this research not only unravels the functional nuances of Cas12j variants, but also contributes to the advancement of Cas12j‐based diagnostic tools, paving the way for their future clinical application.

## 4 Experimental Section

### Ethical Statement

The methodology adopted for this study was reviewed and approved by the Institutional Review Board of Samsung Medical Center (SMC 2021‐06‐083‐015) and written informed consent was obtained from all patients prior to blood sampling. All specimens were anonymized with a unique number and access rights were restricted to only relevant researchers.

### Materials

All oligonucleotides were synthesized and purified by Bioneer (Daejeon, Korea). Custom crRNAs were purchased from IDT (Coralville, IA, USA). Luna Universal One‐Step RT‐qPCR Kit, Vent (exo‐) DNA polymerase, Nt.BstNB I, and dNTP were purchased from New England Biolabs (Ipswich, MA, USA). The plasmids used for protein expression were obtained from Addgene (plasmid # 158794–158796).^[^ ^7^ ^]^ Recombinant RNase inhibitor (RRI) was purchased from Takara Korea Biomedical Inc. (Seoul, Korea). Dulbecco's modified Eagle's medium (DMEM) and Fetalgro Bovine Growth Serum (FBS) were purchased from Welgene Inc. (Gyeongsan, Korea) and RMBIO (Missoula, MT, USA), respectively. A549, WI‐38, DU145, HeLa, PC3, and SK‐BR‐3 cell lines were obtained from Korean Cell Line Bank (Seoul, Korea). Ultrapure DNase/RNase‐free distilled water was purchased from Bioneer and used in all experiments. All other chemicals were analytical grade and used without further purification.

### Protein Expression and Purification

Cas12j variants, tagged at the N‐terminus with a hexa‐histidine sequence, were cloned into the pRSFDuet vector and transformed into *E. coli* RIL cells. The cells were cultured at 37 °C in Luria‐Berani (LB) medium until the optical density at 600 nm (OD_600_) reached 0.5–0.7. Protein overexpression was induced with 1 mM isopropyl β‐D‐1‐thiogalactopyranoside (IPTG) and incubated at 18 °C for 18 h. Cells were harvested, resuspended in lysis buffer (2 M NaCl, 50 mM 4‐(2‐hydroxyethyl)−1‐piperazine ethanesulfonic acid (HEPES), 5 mM 2‐mercaptoethanol, and 5% glycerol) and sonicated. Following centrifugation, the supernatant was diluted 1:1 with a no‐salt buffer (50 mM HEPES, 5 mM 2‐mercaptoethanol, and 5% glycerol). The soluble protein fraction was loaded onto a pre‐equilibrated HisTrap HP 5 mL column (Cytiva, Marlborough, MA, USA) using A buffer (500 mM NaCl, 50 mM HEPES, 5 mM 2‐mercaptoethanol, 5% glycerol, and 20 mM imidazole). Proteins were then eluted with B buffer (500 mM NaCl, 50 mM HEPES, 5 mM 2‐mercaptoethanol, 5% glycerol, and 500 mM imidazole), purified by size exclusion chromatography on a Superdex200 26/600 column (Cytiva), and equilibrated with SEC buffer (500 mM NaCl, 30 mM HEPES, 3 mM 2‐mercaptoethanol, and 5% glycerol). The purified protein fractions were concentrated to 10 mg mL^−1^ using a centrifugal filter (Merck, Darmstadt, Germany), flash‐frozen in liquid nitrogen, and stored at −80 °C.

### RNP Preparation

To facilitate the formation of a hairpin structure, the crRNA was heated to 95 °C for 3 min and allowed to cool to 25 °C. The Cas12j proteins were diluted to a concentration of 40 µM using SEC buffer, and the crRNA was diluted to 40 µM using diethyl pyrocarbonate (DEPC)‐treated water. They were then mixed in a 1:1 ratio and incubated at 25 °C for 30 min.

### In Vitro Trans‐Cleavage Activity Assay

For the in vitro trans‐cleavage activity assay, a 10 µL reaction mixture was prepared, comprising 2 µL each of reaction buffer, cofactor solution, RNP, target DNA, and reporter probe. The final concentrations of RNP, target DNA, and reporter probe were all 250 nM. The mixture was incubated at 37 °C for 30 min. Optimization of assay conditions involved adjusting the pH of the reaction buffer to 7.5‐9.0 using Tris‐HCl, varying NaCl concentrations from 0 to 200 mM, and evaluating cofactors including MgCl₂, CoSO₄, CaCl₂, CuSO₄, and NiCl₂ at concentrations between 0 to 15 mM. DTT concentration was adjusted from 0 to 20 mM, BSA from 0 to 800 µg µL^−1^, and glycerol from 0 to 20%. The assay also examined the effect of target sequence length, using sequences ranging from 9 to 80 nt. Thermal dependency was evaluated between 5 and 65 °C. Specificity was determined by introducing single nucleotide mutations into 20 different target sequences. Following the reaction, samples were diluted 10‐fold with a buffer containing 50 mM NaCl and 10 mM Tris‐HCl at pH 8.5, and fluorescence intensities were measured using a Tecan Infinite M200 Pro set to an excitation wavelength of 488 nm and an emission wavelength of 535 nm with black 96‐well microplates for analysis.

### EXP‐J Reaction for miRNA Detection

For the EXP‐J reaction, a 20 µL reaction solution was prepared with 6.2 µL DEPC‐treated water, 1.5 µL MgSO_4_ (100 mM), 1 µL 10× Thermopol buffer, 0.4 µL 10× NEBuffer 3.1, 5 µL dNTP (2 mM), 1 µL converter (100 nM), 1 µL repeater (200 nM), 0.4 µL RRI (40 U µL^−1^), 0.5 µL Nt.BstNB I (10 U µL^−1^), 1 µL Vent (exo‐) DNA polymerase (2 U µL^−1^), and 2 µL sample solution. The reaction solution was incubated at 55 °C for 30 min. Next, a 5 µL Cas12j reaction solution containing 1 µL 10× NEBuffer 2.1, 1 µL reporter DNA (10 µM), 1 µL Cas12j/crRNA (2.5 µM), and 2 µL MgCl_2_ (87.5 mM) were added to the reaction solution and the fluorescence signal was measured with CFX96 Real‐Time System at 37 °C (Bio‐Rad, Hercules, CA, USA).

For the EXPAR reaction only, 20 µL reaction solution was prepared with 5.2 µL DEPC‐treated water, 1.5 µL MgSO_4_ (100 mM), 1 µL 10× Thermopol buffer, 0.4 µL 10× NEBuffer 3.1, 5 µL dNTP (2 mM), 1 µL converter (100 nM), 1 µL repeater (200 nM), 0.4 µL RRI (40 U µL^−1^), 1 µL 20× SYBR Green I, 0.5 µL Nt.BstNB I (10 U µL^−1^), 1 µL Vent (exo‐) DNA polymerase (2 U µL^−1^), and 2 µL sample solution. The fluorescence signal was measured with CFX96 Real‐Time System at 55 °C (Bio‐Rad).

### Gel electrophoresis analysis

For PAGE analysis, 10 µL reaction solution was reacted for 10 min and mixed with 2 µL orange loading dye (6×, New England Biolabs) and resolved on 10% polyacrylamide gel at a constant voltage of 120 V for 60 min using tris‐borate‐ ethylene‐diamine‐tetra acetic acid (EDTA) (TBE, 1×) as a running buffer. After electrophoresis, the gel was subjected to GelRed solution for staining and images were taken with a GelDoc Imaging System (Bio‐Rad).

### Cell Culture and miRNA Extraction

All cell lines were cultured in DMEM supplemented with 10% FBS under humidified atmosphere containing 5% CO_2_ at 37 °C. During the exponential growth phase, the cells were collected and counted by LUNA‐II automated cell counter (Logos Biosystems Inc., Anyang, Korea). From the 1 × 10^6^ cells, miRNAs were extracted by using the miRNeasy Tissue/Cells Mini Kit (Qiagen, Hilden, Germany) following the instruction of the manufacturer. The concentration of the extracted miRNAs was determined by using a NanoDrop spectrophotometer (Thermo Fisher, Wilmington, DE, USA) and diluted down to 30 ng µL^−1^. Finally, 2 µL of extracted miRNAs was analyzed by EXP‐J reaction.

### EXP‐J Reaction for Lung Cancer Diagnosis

The plasma samples from the patients aged 19 years or older who were diagnosed or suspected of having lung cancer and who had not yet started treatment were obtained at Samsung Medical Center. Less than 20 mL of whole blood was collected in EDTA‐treated vacuum tubes and immediately stored at 4 °C. Within 8 h of collection, plasma was separated by centrifugation (1600 g) at 4 °C for 10 min, placed in a sterilized tube, and stored at −80 °C. The miRNeasy Serum/Plasma Advanced Kit (Qiagen) was utilized to extract the total miRNA in plasma samples from patients or healthy donors (Single Donor Human Plasma (Blood Derived), Innovative Research, Inc., Novi, MI, USA) following the instruction of the manufacturer. Finally, 1 µL of extracted miRNAs was analyzed by EXP‐J reaction.

### PCR

For PCR, conventional stem‐loop qPCR using Luna Universal One‐Step RT‐qPCR Kit was used. In detail, 12 µL solution containing 0.6 µL sample, 0.48 µL stem‐loop primer (10 µM), 0.6 µL forward primer (10 µM), 0.6 µL reverse primer (10 µM), 1× Luna Universal one‐step reaction mix, and 1× Luna WarmStart RT enzyme mix was subjected to RT reaction at 55 °C for 10 min and initial denaturation at 95 °C for 1 min, followed by 45 cycles of 10 s at 95 °C, and 30 s at 60 °C.^[^ ^27^ ^]^ The fluorescent signal was measured every cycle with CFX96 Real‐Time System.

### Statistical Analysis

No statistical method was used to predetermine sample size, but our sample sizes are similar to those reported in previous publications.^[^ ^28^ ^]^ Data distribution was assumed to be normal, but this was not formally tested. No data were excluded from the analyses. Statistical tests through the manuscript were performed using Prism 10 (GraphPad). Intergroup differences were analyzed by unpaired, two‐tailed Student's t‐test with Welch correction for single comparison. Statistical significance was considered as p < 0.05. All data are presented as mean ± SD.

## Conflict of Interest

The authors declare no conflict of interest.

## Author Contributions

J.‐E.K., H.K., and Y.‐H.L. contributed equally to this work. J.‐E.K., Y.‐H.L., H.‐Y.L., and J.‐R.K conceived, designed, and performed experiments on Cas12j variants and contributed to writing the manuscript. H.K. conceived, designed, and performed experiments on EXP‐J reactions and wrote the manuscript. Y.P. and H.J. assisted the miRNA expression analysis and were involved in discussion. M.‐Y.L. and B.‐H.J. provided clinical samples from lung cancer patients. J.‐Y.B., S.J.K., E.‐K.L., and J.J. were involved in the design of the experiments and in discussion. E.‐J.W., T.K., and K.‐H.P. conceived and supervised the research, wrote the paper, and acquired funding. All authors reviewed and provided feedback on the manuscript.

## Supporting information

Supporting Information

## Data Availability

The data that support the findings of this study are available in the supplementary material of this article.
