# Supplementary material for: Unveiling Cas12j Trans‐Cleavage Activity for CRISPR Diagnostics: Application to miRNA Detection in Lung Cancer Diagnosis
Source: Adv Sci (Weinh). 2024 Oct 1;11(44):2402580. doi: 10.1002/advs.202402580 (PMC11600238; doi:10.1002/advs.202402580)
Supplement: Supplementary file 1 — Supporting Information [file ADVS-11-2402580-s001.docx]

Supplementary Information

**Unveiling Cas12j Trans-Cleavage Activity for CRISPR Diagnostics: Application to miRNA Detection in Lung Cancer Diagnosis**

Ju-Eun Kang^1,2,†^, Hansol Kim^3,†^, Young-Hoon Lee^1,2,†^, Ha-Yeong Lee^1^, Yeonkyung Park^3^, Hyowon Jang^3^, Jae-Rin Kim^1,2^, Min-Young Lee^4^, Byeong-Ho Jeong^5^, Ju-Young Byun^1,3^, Seung Jun Kim^1^, Eun-Kyung Lim^3,6,7^, Juyeon Jung^3,6,7^, Eui-Jeon Woo^1,2,8,*^, Taejoon Kang^3,6*^, and Kwang-Hyun Park^1,2,8*^

^1^Critical Diseases Diagnostics Convergence Research Center, Korea Research Institute of Bioscience and Biotechnology (KRIBB), Daejeon 34141, Republic of Korea

^2^Department of Proteome Structural Biology, KRIBB School of Bioscience, University of Science and Technology (UST), Daejeon 34113, Republic of Korea

^3^Bionanotechnology Research Center, KRIBB, Daejeon 34141, Republic of Korea

^4^Department of Nano-Bio Convergence, Surface Materials Division, Korea Institute of Materials Science (KIMS), Changwon, Gyeongsangnam-do 51508, Republic of Korea

^5^Division of Pulmonary and Critical Care Medicine, Department of Medicine, Samsung Medical Center, Sungkyunkwan University (SKKU) School of Medicine, Seoul 06351, Republic of Korea

^6^School of Pharmacy, SKKU, Suwon, Gyeongi-do 16419, Republic of Korea

^7^Department of Nanobiotechnology, KRIBB School of Biotechnology, UST, Daejeon 34113, Republic of Korea

^8^Disease Target Structure Research Center, KRIBB, Daejeon 34141, Republic of Korea

†These authors contributed equally to this work

*Corresponding author: ruuua@kribb.re.kr (K.-H.P.), kangtaejoon@kribb.re.kr (T.K.), ejwoo@kribb.re.kr (E.-J.W.)


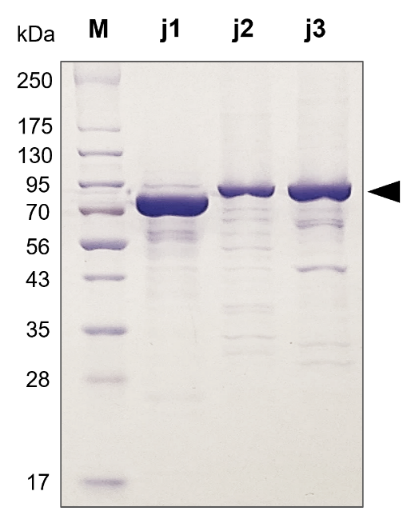


**Figure S1. SDS-PAGE analysis of purified Cas12j variants.** The SDS-PAGE was used to assess the purity of three Cas12j variants (Cas12j1 in lane j1, Cas12j2 in lane j2, and Cas12j3 in lane j3). Lane M contains the molecular weight marker, with sizes indicated in kDa on the left. The arrowhead marks the position of Cas12j proteins. The theoretical molecular weights are approximately 82 kDa for Cas12j1, 88 kDa for Cas12j2, and 89 kDa for Cas12j3.


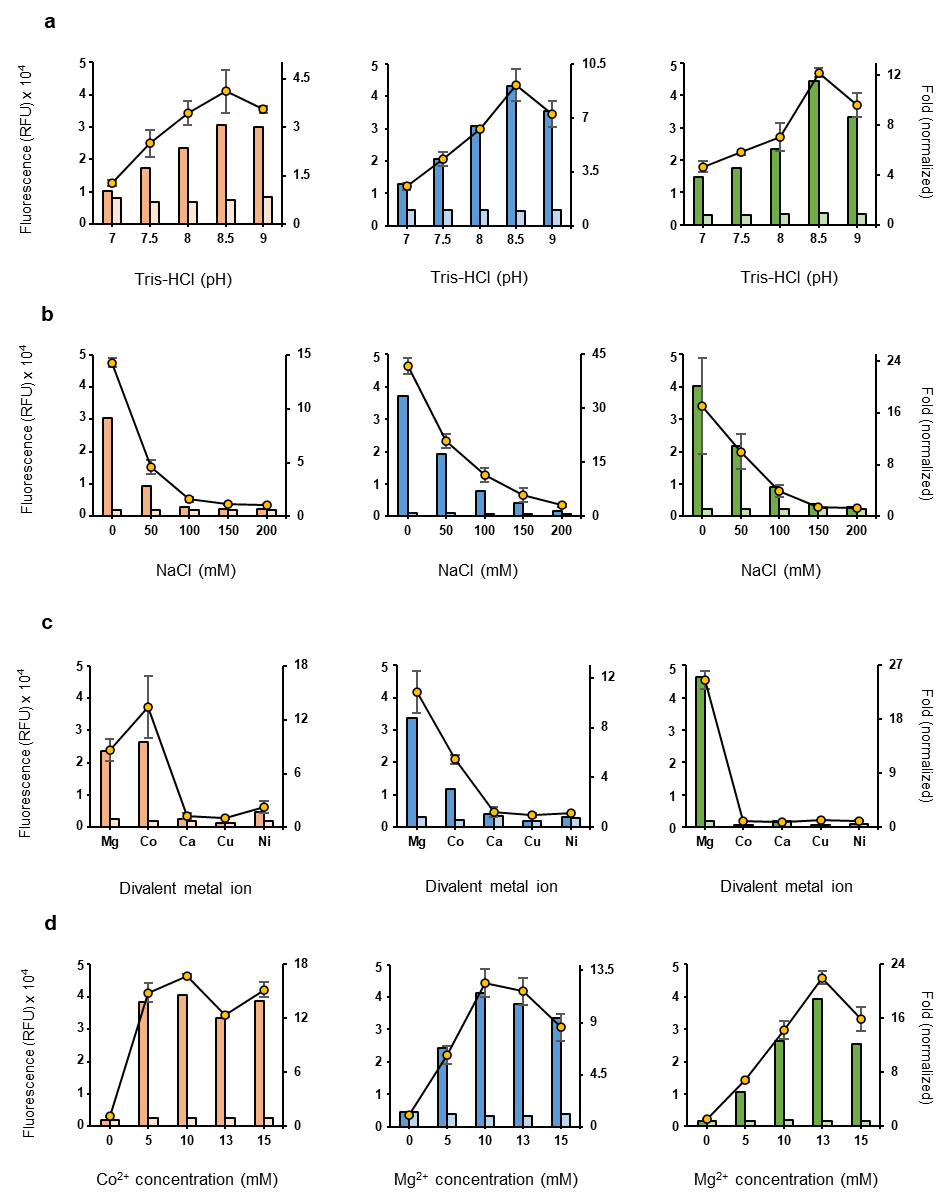


**Figure S2. Effect of buffer conditions on Cas12j trans-cleavage efficiency.** Evaluation of trans-cleavage activity for three Cas12j variants (Cas12j1 in orange, Cas12j2 in blue, and Cas12j3 in green) in the presence (dark bars) or absence (light bars) of ssDNA target under different buffer conditions. Factors evaluated include (**a**) pH variation, (**b**) salt concentration, (**c**) specific metal ion, and (**d**) divalent metal ion concentration. Fluorescence intensities are shown in the bar graph (left *y*-axis). Fold-changes, normalized to samples without target, are plotted in the line graph (right *y*-axis). Error bars indicate SD of three replicates.


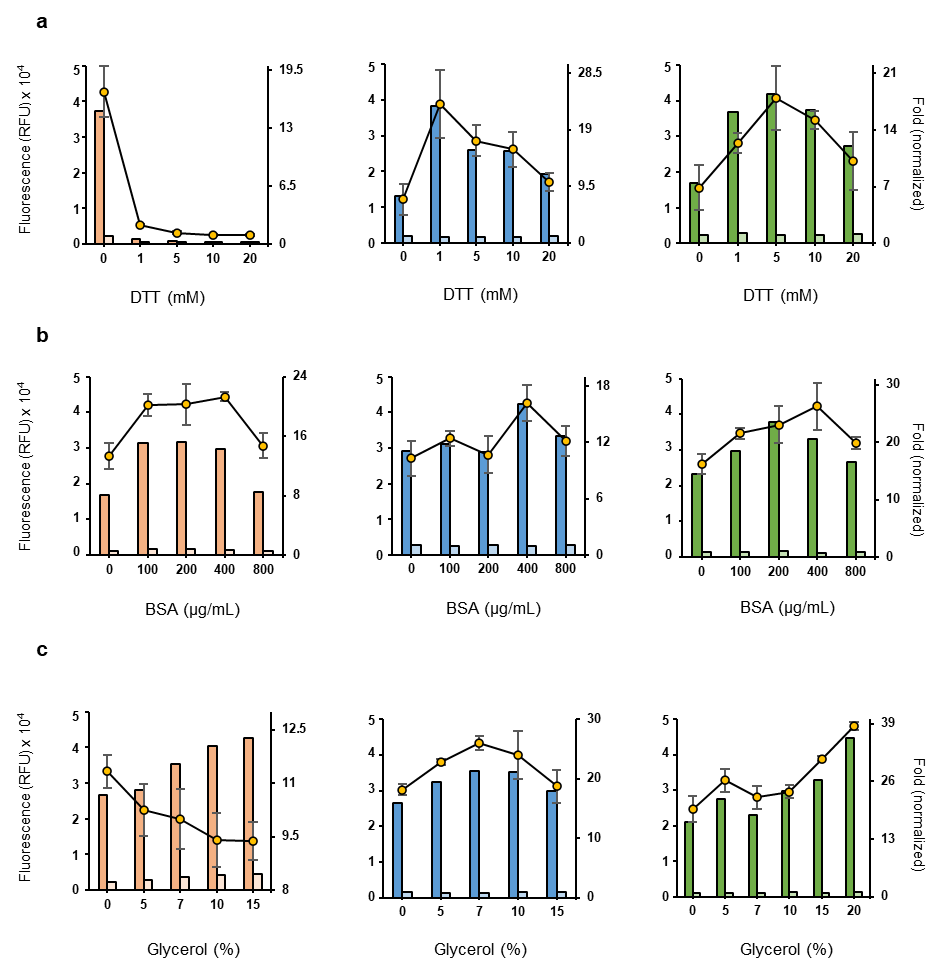


**Figure S3. Effect of additives on Cas12j trans-cleavage efficiency.** Evaluation of trans-cleavage activity for three Cas12j variants (Cas12j1 in orange, Cas12j2 in blue, and Cas12j3 in green) in the presence (dark bars) or absence (light bars) of ssDNA target under different additives in buffer. Factors evaluated include (**a**) DTT concentration, (**b**) BSA concentration, and (**c**) glycerol concentration. Fluorescence intensities are shown in the bar graph (left *y*-axis). Fold-changes, normalized to samples without target, are plotted in the line graph (right *y*-axis). Error bars indicate SD of three replicates.


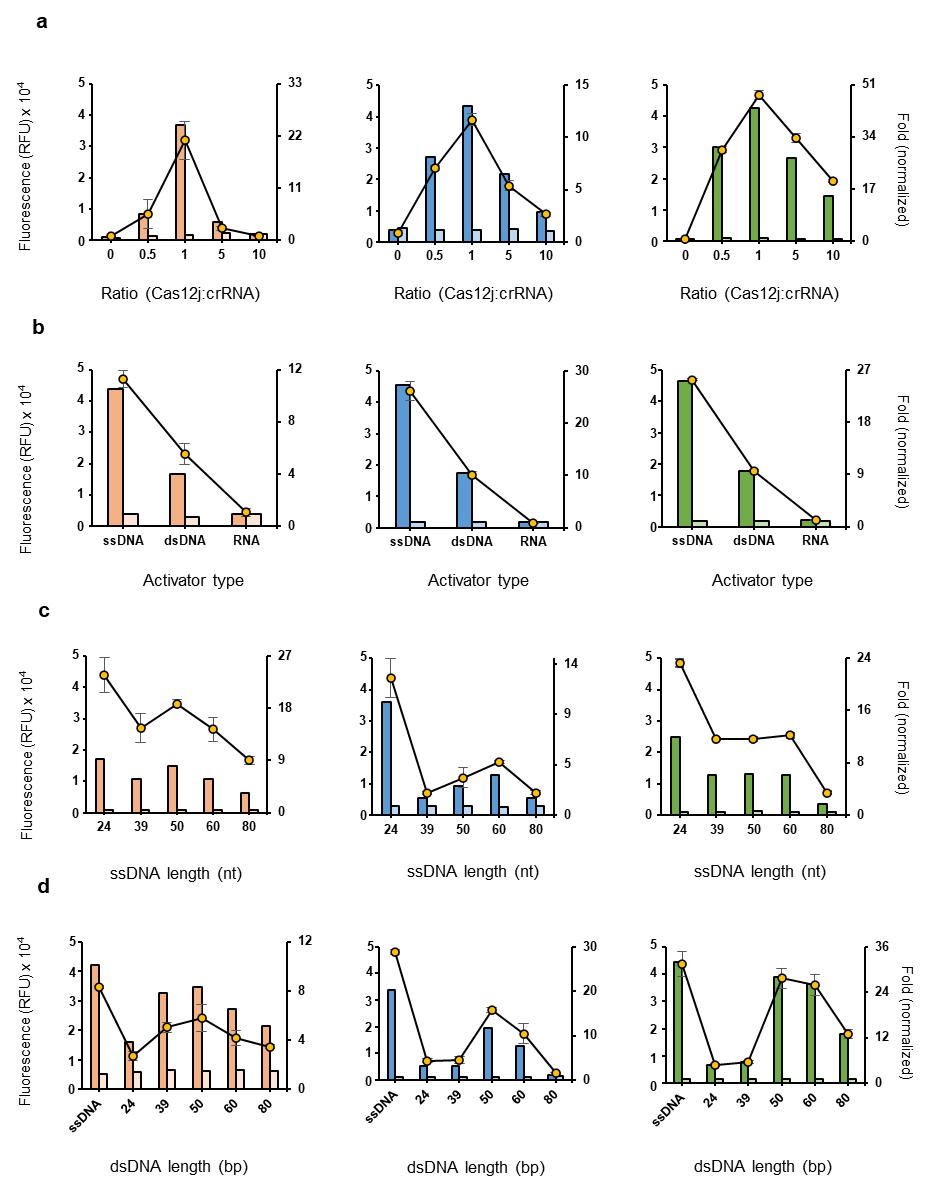


**Figure S4. Effect of crRNA concentration and activator type and length on Cas12j trans-cleavage efficiency.** Evaluation of trans-cleavage activity for three Cas12j variants (Cas12j1 in orange, Cas12j2 in blue, and Cas12j3 in green) in the presence (dark bars) or absence (light bars) of target under different parameters. Factors evaluated include (**a**) crRNA concentration, (**b**) Activator type, and length of (**c**) ssDNA and (**d**) dsDNA. Fluorescence intensities are shown in the bar graph (left *y*-axis). Fold-changes, normalized to samples without target, are plotted in the line graph (right *y*-axis). Error bars indicate SD of three replicates.

**
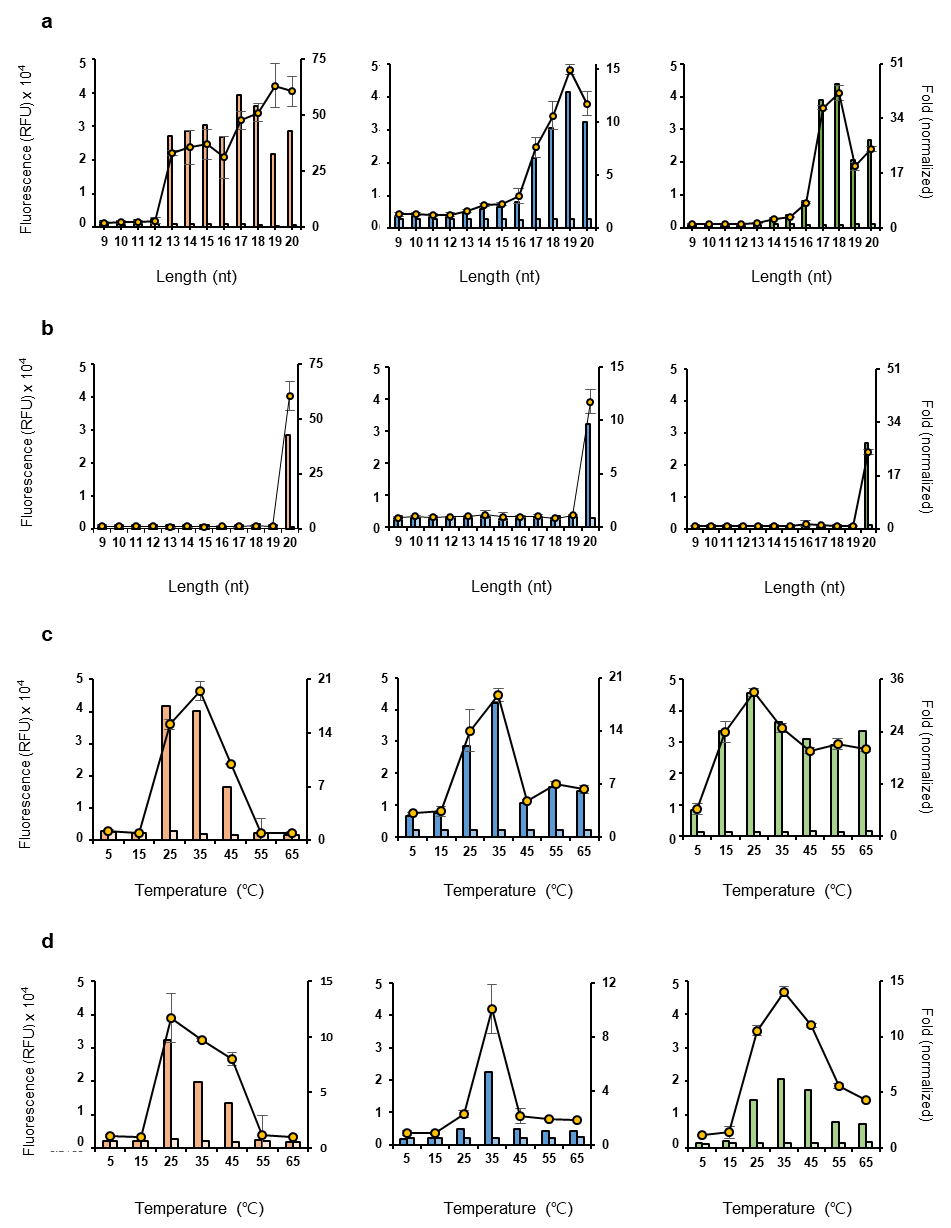
**

**Figure S5. Effect of ssDNA target length and temperature on Cas12j trans-cleavage efficiency.** Evaluation of trans-cleavage for three Cas12j variants (Cas12j1 in orange, Cas12j2 in blue, and Cas12j3 in green) in the presence (dark bars) or absence (light bars) of target under different parameters. Factors evaluated include (**a**) ssDNA target length from the 3' end, (**b**) ssDNA target length from the 5' end, and temperature using (**c**) ssDNA and (**d**) dsDNA targets. Fluorescence intensities are shown in the bar graph (left *y*-axis). Fold-changes, normalized to samples without target, are plotted in the line graph (right *y*-axis). Error bars indicate SD of three replicates.


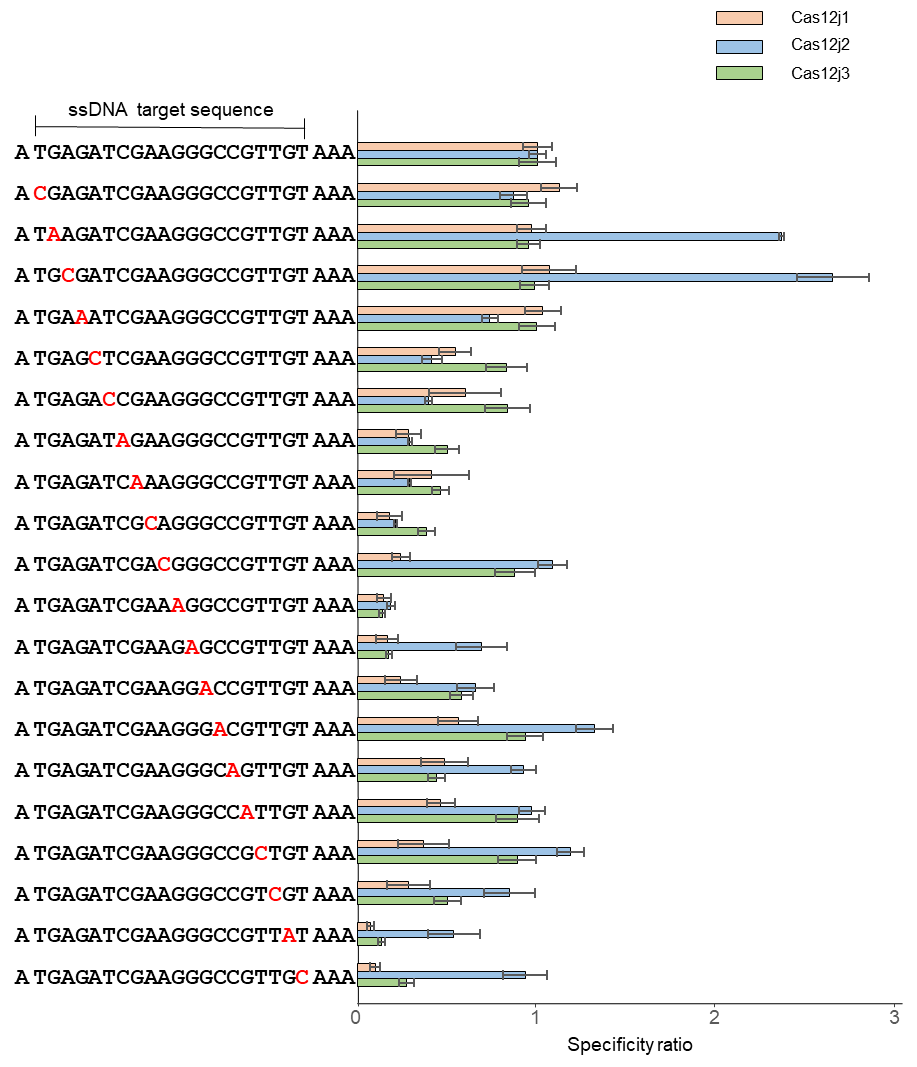


**Figure S6. Specificity of Cas12j trans-cleavage activity against mismatched ssDNA targets.** Depicted sequences show PM and mismatches highlighted in bold red. Specificity ratio is shown in the bar graph. Data are normalized to fold-changes with respect to PM targets. Error bars indicate SD of7 three replicates.


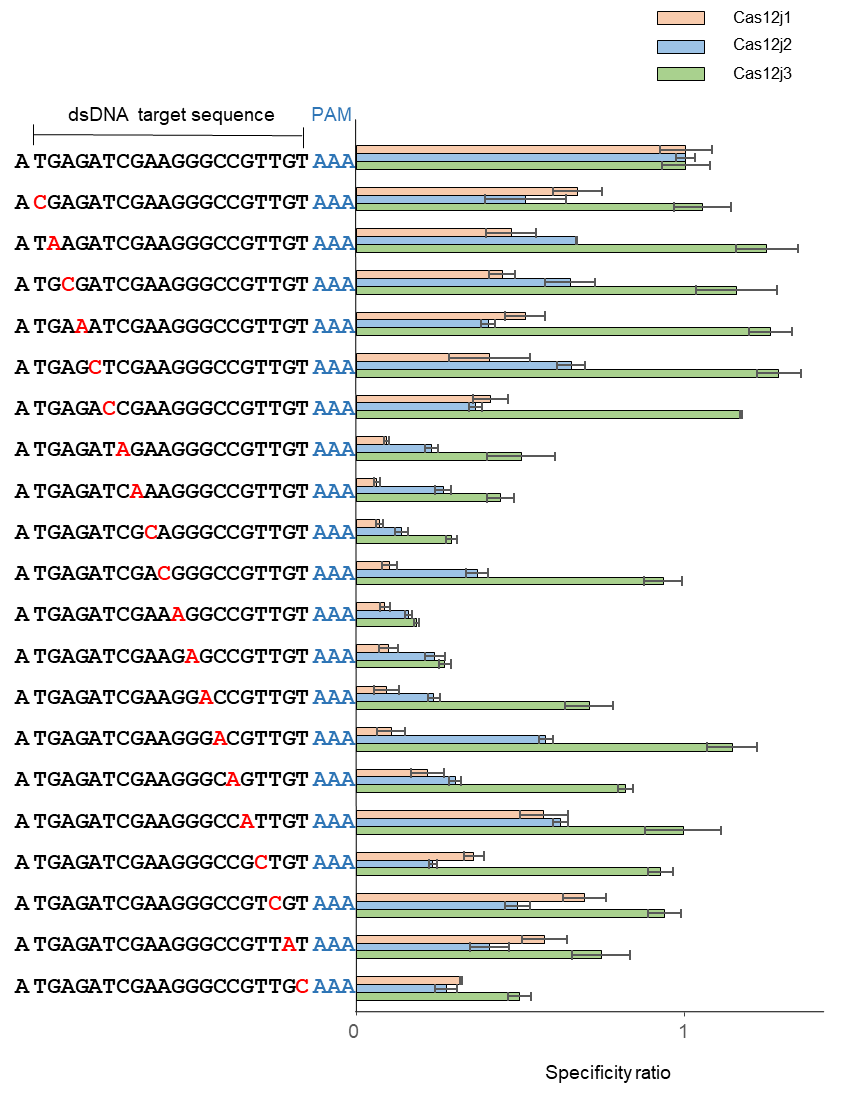


**Figure S7. Specificity of Cas12j trans-cleavage activity against mismatched dsDNA targets.** Depicted sequences show PM and mismatches highlighted in bold red. Specificity ratio is shown in the bar graph. Data are normalized to fold-changes with respect to PM targets. Error bars indicate SD of three replicates.


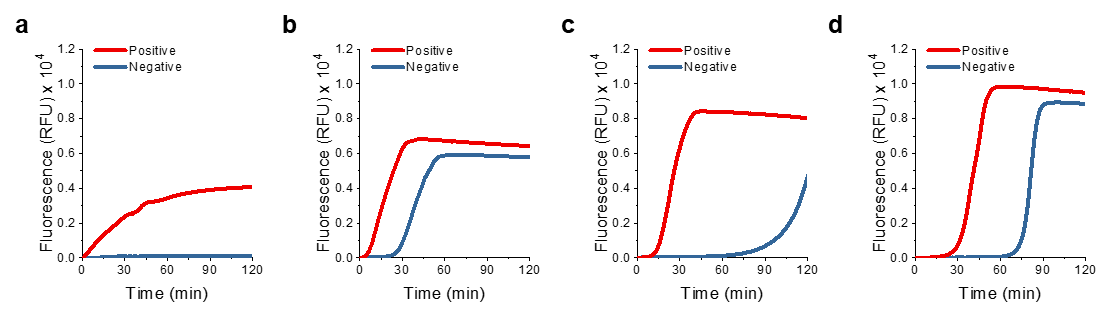


**Figure S8. Optimization of the EXP-J reaction temperature.** Real-time fluorescence curves obtained from the EXPAR reaction with (red) and without (blue) 100 pM trigger. The reaction temperatures were varied at (**a**) 64.5°C, (**b**) 59°C, (**c**) 55°C, and (**d**) 52°C. Optimization was performed on the EXPAR reaction without the Cas12j and independently replicated three times. In detail, 20 μL reaction solution was prepared with 6.2 μL DEPC-treated water, 1.5 μL MgSO_4_ (100 mM), 1 μL 10× Thermopol buffer, 0.4 μL 10× NEBuffer 3.1, 5 μL dNTP (2 mM), 1 μL repeater (2 μM), 0.4 μL RRI (40 U/μL), 1 μL 20× SYBR Green I, 1 μL Vent (exo-) DNA polymerase (2 U/μL), 0.5 μL Nt.BstNB I (10 U/μL), and 2 μL trigger (1 nM). The reaction temperature of 55°C was selected as optimal and used for further experiments.


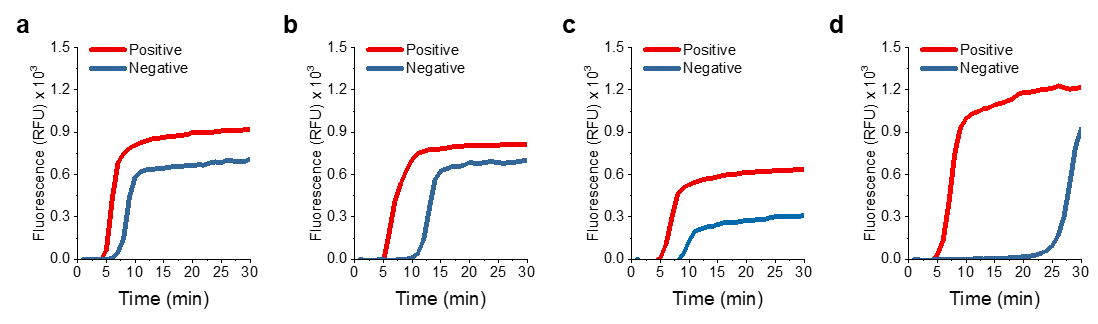


**Figure S9. Optimization of trigger length in the EXP-J reaction.** Real-time fluorescence curves obtained from the EXPAR reaction with (red) and without (blue) 1 nM miR-21. The trigger lengths of the repeater were varied for (**a**) 16 nt, (**b**) 17 nt, (**c**) 18 nt, and (**d**) 19 nt. Optimization was performed on the EXPAR reaction without the Cas12j and independently replicated three times. In detail, 20 μL reaction solution was prepared with 5.2 μL DEPC-treated water, 1.5 μL MgSO_4_ (100 mM), 1 μL 10× Thermopol buffer, 0.4 μL 10× NEBuffer 3.1, 5 μL dNTP (2 mM), 1 μL converter (100 nM), 1 μL repeater (200 nM), 0.4 μL RRI (40 U/μL), 1 μL 20× SYBR Green I, 1 μL Vent (exo-) DNA polymerase (2 U/μL), 0.5 μL Nt.BstNB I (10 U/μL), and 2 μL miR-21 (10 nM). The trigger length of 19 nt was selected as optimal and used for further experiments.

**
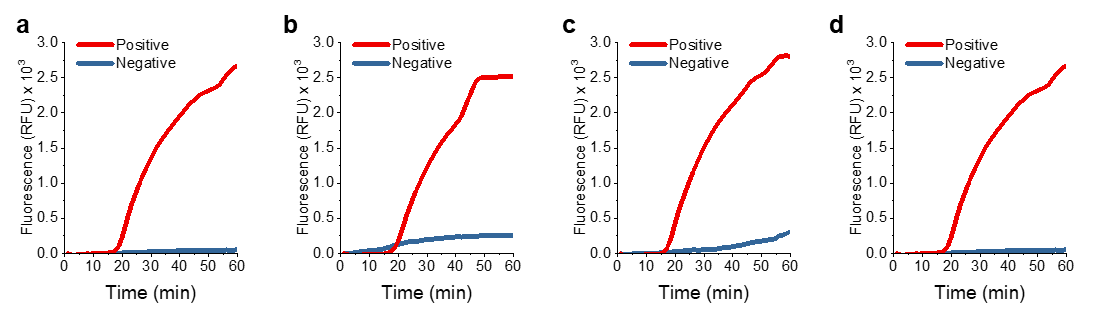
**

**Figure S10. Optimization of converter concentration in the EXP-J reaction.** Real-time fluorescence curves obtained from the EXPAR reaction with (red) and without (blue) 10 pM miR-21. The converter-21 concentrations were varied for (**a**) 0.5 nM, (**b**) 1 nM, (**c**) 2 nM, and (**d**) 5 nM. Optimization was performed on the EXPAR reaction without the Cas12j and independently replicated three times. In detail, 20 μL reaction solution was prepared with 5.2 μL DEPC-treated water, 1.5 μL MgSO_4_ (100 mM), 1 μL 10× Thermopol buffer, 0.4 μL 10× NEBuffer 3.1, 5 μL dNTP (2 mM), 1 μL repeater (200 nM), 0.4 μL RRI (40 U/μL), 1 μL 20× SYBR Green I, 1 μL Vent (exo-) DNA polymerase (2 U/μL), 0.5 μL Nt.BstNB I (10 U/μL), 2 μL miR-21 (10 nM), and 1 μL converter-21 with various concentrations. The converter concentration of 5 nM was selected as optimal and used for further experiments.


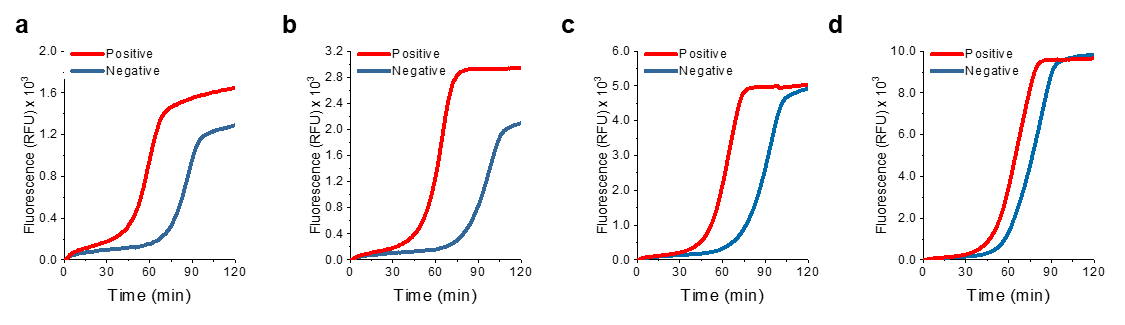


**Figure S11. Optimization of repeater concentration in the EXP-J reaction.** Real-time fluorescence curves obtained from the EXPAR reaction with (red) and without (blue) 10 pM miR-21. The repeater concentrations were varied for (**a**) 5 nM, (**b**) 10 nM, (**c**) 20 nM, and (**d**) 50 nM. Optimization was performed on the EXPAR reaction without the Cas12j and independently replicated three times. In detail, 20 μL reaction solution was prepared with 5.2 μL DEPC-treated water, 1.5 μL MgSO_4_ (100 mM), 1 μL 10× Thermopol buffer, 0.4 μL 10× NEBuffer 3.1, 5 μL dNTP (2 mM), 1 μL converter-21 (100 nM), 0.4 μL RRI (40 U/μL), 1 μL 20× SYBR Green I, 1 μL Vent (exo-) DNA polymerase (2 U/μL), 0.5 μL Nt.BstNB I (10 U/μL), 2 μL miR-21 (10 nM), and 1 μL repeater with various concentrations. The repeater concentration of 10 nM was selected as optimal and used for further experiments.


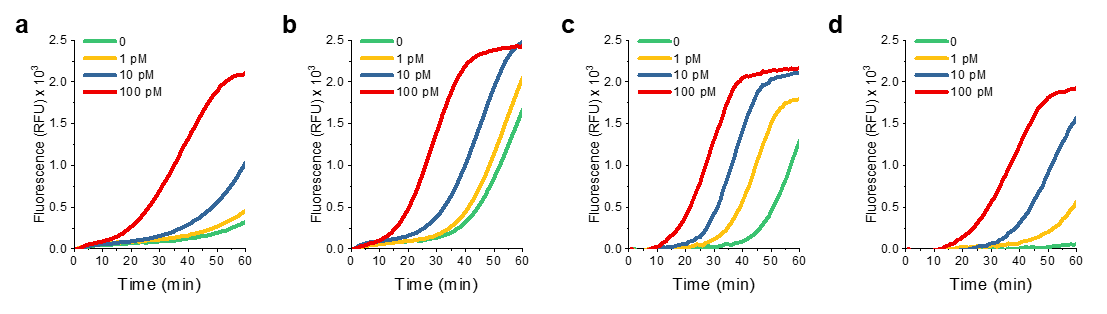


**Figure S12. Optimization of rNEBuffer 3.1 concentration in the EXP-J reaction.** Real-time fluorescence curves obtained from the EXPAR reaction with various concentrations of miR-21 (0, 1, 10, and 100 pM). The rNEBuffer 3.1 concentrations were varied for (**a**) 0×, (**b**) 0.1×, (**c**) 0.2×, and (**d**) 0.4×. Optimization was performed on the EXPAR reaction without the Cas12j and independently replicated three times. In detail, 20 μL reaction solution was prepared with DEPC-treated water, 1.5 μL MgSO_4_ (100 mM), 1 μL 10× Thermopol buffer, 5 μL dNTP (2 mM), 1 μL converter-21 (100 nM), 1 μL repeater (200 nM), 0.4 μL RRI (40 U/μL), 1 μL 20× SYBR Green I, 1 μL Vent (exo-) DNA polymerase (2 U/μL), 0.5 μL Nt.BstNB I (10 U/μL), 2 μL miR-21 (10 nM), and 10× NEBuffer 3.1 with various volumes (0, 0.2, 0.4, and 0.8 μL). The rNEBuffer 3.1 concentration of 0.2× was selected as optimal and used for further experiments.

**Figure S13. Optimization of Cas12j3/crRNA concentration in the EXP-J reaction.** Fluorescence intensities obtained from the EXP-J reaction with (red) and without (blue) 1 nM miR-21. The Cas12j3/crRNA concentrations were varied for 20 nM, 50 nM, 100 nM, and 200 nM, while the EXPAR reaction conditions were maintained at the previously optimized conditions. The mean fluorescence intensities from three technical replicates are represented as bars and individual dots indicate raw fluorescence data from each experiment. Error bars indicate SD. The final concentration of reporter DNA is 400 nM. The Cas12j3/crRNA concentration of 100 nM was selected as optimal and used for further experiments.


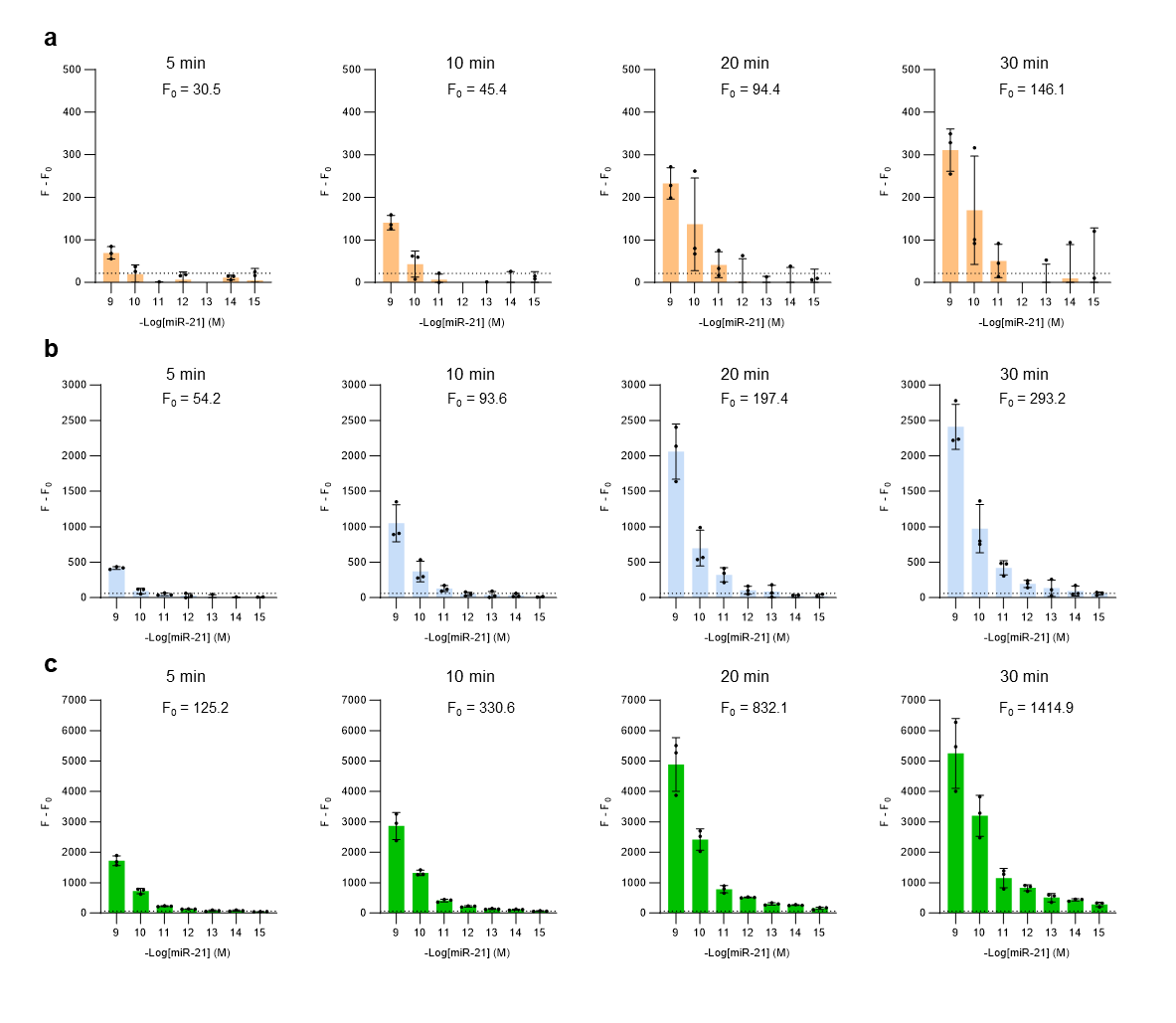


**Figure S14. EXP-J reaction for miRNA detection.** Comparison of EXP-J reaction efficiency using (**a**) Cas12j1, (**b**) Cas12j2, and (**c**) Cas12j3, respectively. EXP-J reactions were performed with different Cas12j reaction times and miR-21 concentrations. Dotted line represents the threshold value (F - F_0_ + 3SD of blank samples). F and F_0_ represent the fluorescence intensities after EXP-J reactions with and without miR-21 from three technical replicates. F – F_0_ is represented as bars and individual dots indicate raw data from each experiment. Error bars indicate SD. F_0_ values for each time points were indicated on the graphs. The final concentrations of converter-21, repeater, DP, NE, Cas12j/crRNA, and reporter DNA are 5 nM, 10 nM, 0.1 U/μL, 0.25 U/μL, 100 nM, and 400 nM, respectively.


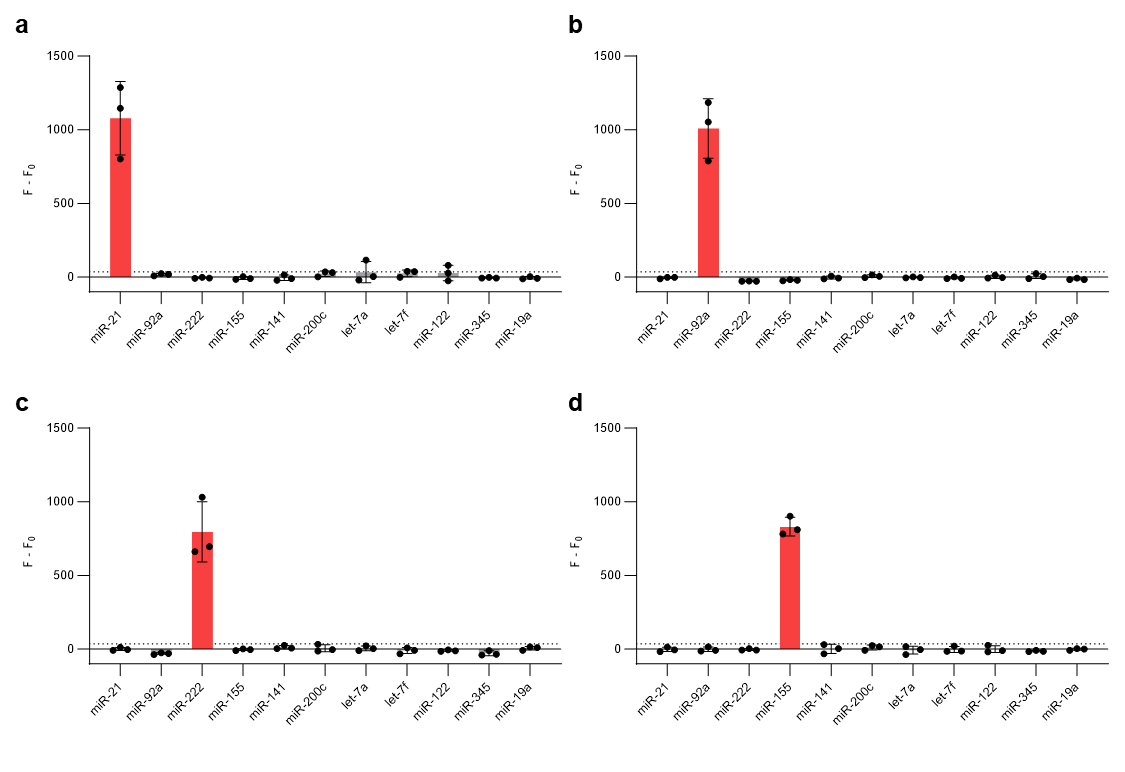


**Figure S15. Selectivity and versatility of the EXP-J reaction.** Plots shows F - F_0_ values of each EXP-J reaction targeting (**a**) miR-21, (**b**) miR-92a, (**c**) miR-222, and (**d**) miR-155) in the presence of various miRNAs. Strong fluorescence signal was observed only when the corresponding miRNA to the target of EXP-J reaction was present. F and F_0_ represent the fluorescence intensities after EXP-J reactions with and without miRNAs from three technical replicates. F – F_0_ is represented as bars and individual dots indicate raw data from each experiment. Error bars indicate SD. The final concentrations of converters, repeater, miRNAs, DP, NE, Cas12j/crRNA, and reporter DNA are 5 nM, 10 nM, 100 pM, 0.1 U/μL, 0.25 U/μL, 100 nM, and 400 nM, respectively.

**
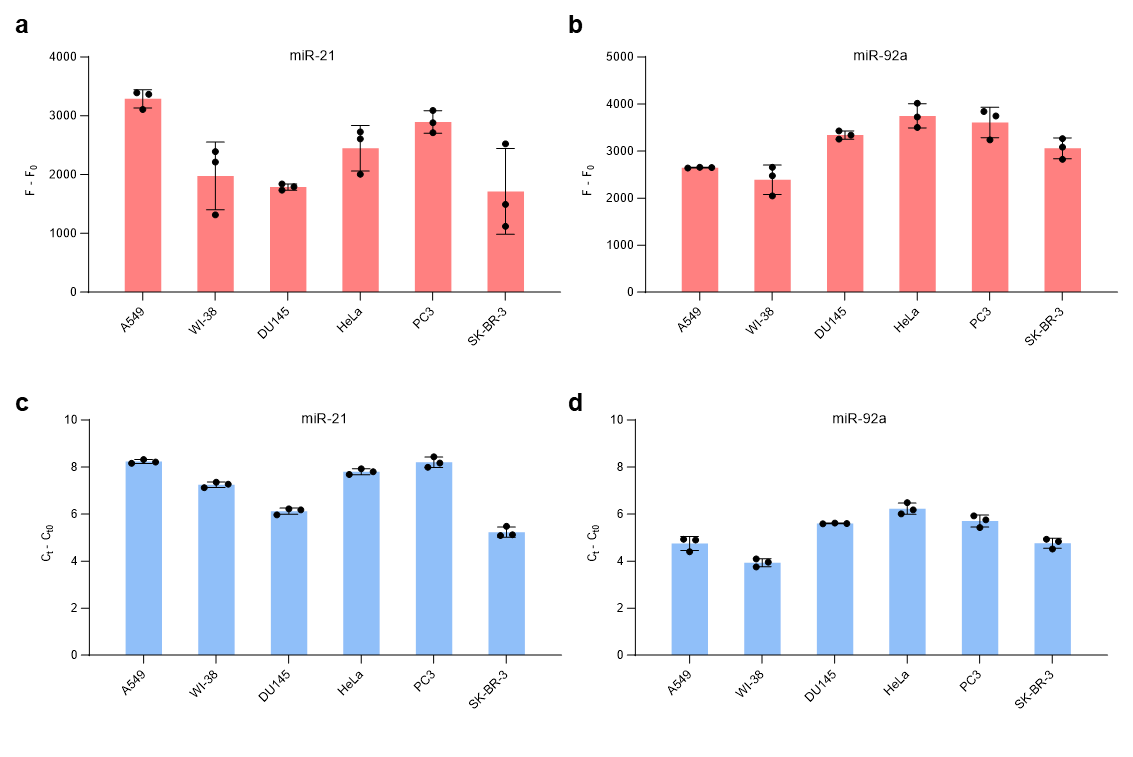
**

**Figure S16. Expression patterns of miR-21 and miR-92a in cell lines using the EXP-J assays and RT-qPCR.** Plots shows F - F_0_ values of each EXP-J reaction targeting (**a**) miR-21 and (**b**) miR-92a with six cell lines. F and F_0_ represent the fluorescence intensities after EXP-J reactions with sample and blank control from three technical replicates. F – F_0_ is represented as bars and individual dots indicate raw data from each experiment. Error bars indicate SD. Plots shows C_t_ – C_t0_ values of RT-qPCR for (**c**) miR-21 and (**d**) miR-92a with six cell lines. C_t_ and C_t0_ represent the cycle threshold with sample and blank control from three technical replicates. C_t_ – C_t0_ is represented as bars and individual dots indicate raw data from each experiment. Error bars indicate SD.


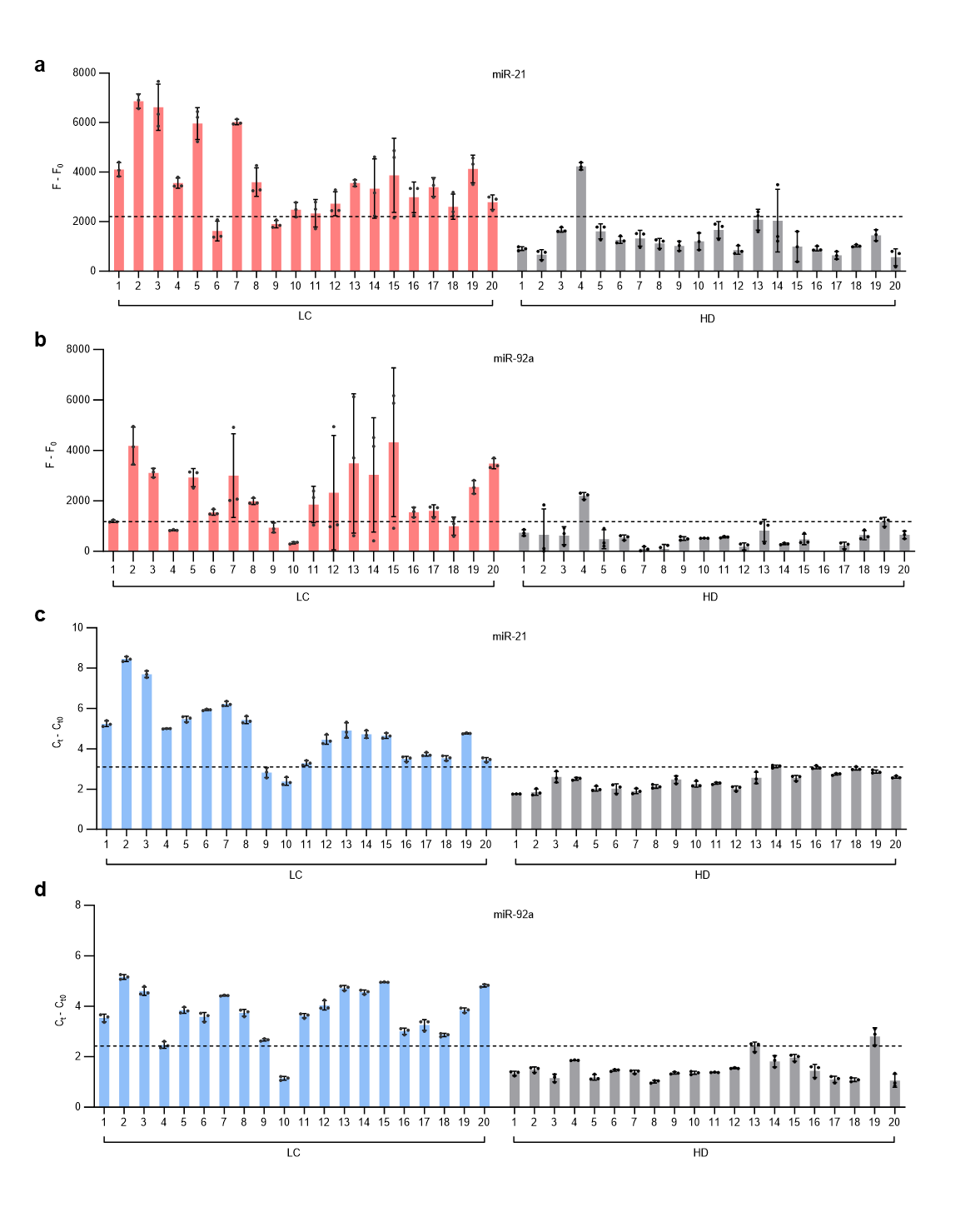


**Figure S17. Analysis of miR-21 and miR-92a from clinical samples using the EXP-J assays and RT-qPCR.** Plots shows F - F_0_ values of each EXP-J reaction targeting (**a**) miR-21 and (**b**) miR-92a with plasma samples of lung cancer patients and healthy controls. F and F_0_ represent the fluorescence intensities after EXP-J reactions with sample and blank control from three technical replicates. F – F_0_ is represented as bars and individual dots indicate raw data from each experiment. Error bars indicate SD. The cut-off lines were determined to be 2209 for miR-21 and 1182 for miR-92, respectively. Plots shows C_t_ – C_t0_ values of RT-qPCR for (**c**) miR-21 and (**d**) miR-92a with plasma samples of lung cancer patients and healthy controls. C_t_ and C_t0_ represent the cycle threshold with sample and blank control from three technical replicates. C_t_ – C_t0_ is represented as bars and individual dots indicate raw data from each experiment. Error bars indicate SD. The cut-off lines were determined to be 3.10 for miR-21 and 2.34 for miR-92a, respectively.

**
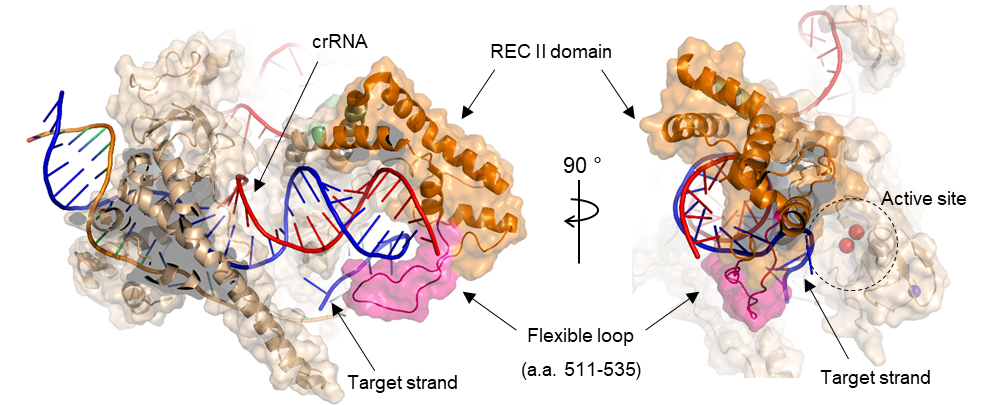
**

**Figure S18. Overall structure of Cas12j2/crRNA with target DNA.** The Cas12j2 structure is shown with a semi-transparent surface to emphasize its secondary structure (PDB ID:7LYT). The nucleic acids are shown in cartoon format to clearly distinguish them. The structure is presented in two orientations. The surface diagram uses a color-coding scheme for component identification: the crRNA is shown in red, the target DNA strand in blue, the REC II domain in orange, and the flexible loop in pink. The images were generated using PyMOL 2.5.


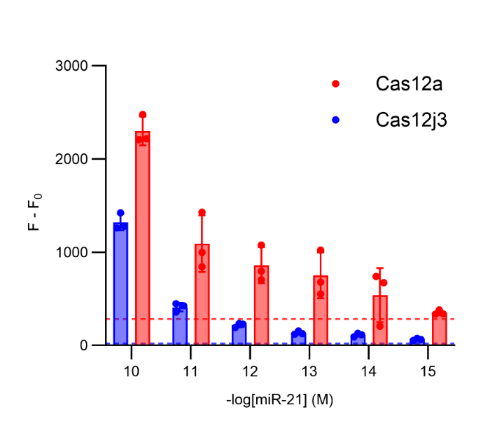
**Figure S19. Comparison of EXP-J reaction efficiency using Cas12a (Alt-R™ A.s. Cas12a (Cpf1) Ultra, IDT, catalog # 10001272) and Cas12j3.** EXP-J reactions were performed under final optimized conditions with different miR-21 concentrations. Dotted line represents the threshold value (F - F_0_ + 3SD of blank samples). F and F_0_ represent the fluorescence intensities after EXP-J reactions with and without miR-21 from three technical replicates. F - F_0_ is represented as bars and individual dots indicate raw data from each experiment. Error bars indicate SD.

**Table S1. Plasmid**

| **Purpose** | **Features** | **Selection marker** |
| --- | --- | --- |
| Protein purification | pRSF-Duet1 derived plasmid containing C-terminally hexa-histidine tagged Cas12j1 in MCS1. (pPP076) | Kanamycin |
| Protein purification | pRSF-Duet1 derived plasmid containing C-terminally hexa-histidine tagged Cas12j2 in MCS1. (pPP085) | Kanamycin |
| Protein purification | pRSF-Duet1 derived plasmid containing C-terminally hexa-histidine tagged Cas12j3 in MCS1. (pPP089) | Kanamycin |

**Table S2. RNA oligonucleotides**

| **Purpose** | **Description** | **Sequence (5' 🡪 3')** |
| --- | --- | --- |
| Trans-cleavage assay crRNA | Sequence contains 25 nt of the 3'-repeat fraction (blue) from the genomic Cas12j1 CRISPR array, followed by a 20 nt spacer corresponding to the target sequence | **AAACGAUUGCUCGAUUAGUCGAGACACAACGGCCCUUCGAUCUCA** |
| Trans-cleavage assay crRNA | Sequence contains 25 nt of the 3'-repeat fraction (blue) from the genomic Cas12j2 CRISPR array, followed by a 20 nt spacer corresponding to the target sequence | **CAACGAUUGCCCCUCACGAGGGGACACAACGGCCCUUCGAUCUCA** |
| Trans-cleavage assay crRNA | Sequence contains 25 nt of the 3'-repeat fraction (blue) from the genomic Cas12j3 CRISPR array, followed by a 20 nt spacer corresponding to the target sequence | **UAUUGAUUGCCCAGUACGCUGGGACACAACGGCCCUUCGAUCUCA** |
| Trans-cleavage assay target | Trans-cleavage RNA substrate with a length of 24 nt (target region shown in blue) | **AUGAGAUCGAAGGGCCGUUGUAAA** |

**Table S3. dsDNA oligonucleotide**

| **Purpose** | **Description** | **Sequence (5' 🡪 3')** |
| --- | --- | --- |
| Trans-cleavage assay target | Trans-cleavage dsDNA substrate with a length of 24 bp (target region shown in blue) | **ATGAGATCGAAGGGCCGTTGTAAA** |
| Trans-cleavage assay target length | Trans-cleavage dsDNA substrate with a length of 39 bp (target region shown in blue) | **TCATGACAAATGAGATCGAAGGGCCGTTGTAAAGCGCCA** |
| Trans-cleavage assay target length | Trans-cleavage dsDNA substrate with a length of 50 bp (target region shown in blue) | **GCTGCTCATGACAATGAGATCGAAGGGCCGTTGTAAAGCGCCAGACTTG** |
| Trans-cleavage assay target length | Trans-cleavage dsDNA substrate with a length of 60 bp (target region shown in blue) | **GCTAGGCTGCTCATGACAAATGAGATCGAAGGGCCGTTGTAAAGCGCCAGACTTGTAAGC** |
| Trans-cleavage assay target length | Trans-cleavage dsDNA substrate with a length of 80 bp (target region shown in blue) | **TAATACGACTCACTATAGGGTCTAGGGACCGTGGCTCGAGCTCATGACAAATGAGATCGAAGGGCCGTTGTAAAGCGCCA** |
| Trans-cleavage assay mismatch tolerance | Mismatched dsDNA substrate (target region shown in blue) mutation position 20 (red) | **GCTGCTCATGACAAACGAGATCGAAGGGCCGTTGTAAAGCGCCAGACTTG** |
| Trans-cleavage assay mismatch tolerance | Mismatched dsDNA substrate (target region shown in blue) mutation position 19 (red) | **GCTGCTCATGACAAATAAGATCGAAGGGCCGTTGTAAAGCGCCAGACTTG** |
| Trans-cleavage assay mismatch tolerance | Mismatched dsDNA substrate (target region shown in blue) mutation position 18 (red) | **GCTGCTCATGACAAATGCGATCGAAGGGCCGTTGTAAAGCGCCAGACTTG** |
| Trans-cleavage assay mismatch tolerance | Mismatched dsDNA substrate (target region shown in blue) mutation position 17 (red) | **GCTGCTCATGACAAATGAAATCGAAGGGCCGTTGTAAAGCGCCAGACTTG** |
| Trans-cleavage assay mismatch tolerance | Mismatched dsDNA substrate (target region shown in blue) mutation position 16 (red) | **GCTGCTCATGACAAATGAGCTCGAAGGGCCGTTGTAAAGCGCCAGACTTG** |
| Trans-cleavage assay mismatch tolerance | Mismatched dsDNA substrate (target region shown in blue) mutation position 15 (red) | **GCTGCTCATGACAAATGAGACCGAAGGGCCGTTGTAAAGCGCCAGACTTG** |
| Trans-cleavage assay mismatch tolerance | Mismatched dsDNA substrate (target region shown in blue) mutation position 14 (red) | **GCTGCTCATGACAAATGAGATAGAAGGGCCGTTGTAAAGCGCCAGACTTG** |
| Trans-cleavage assay mismatch tolerance | Mismatched dsDNA substrate (target region shown in blue) mutation position 13 (red) | **GCTGCTCATGACAAATGAGATCAAAGGGCCGTTGTAAAGCGCCAGACTTG** |
| Trans-cleavage assay mismatch tolerance | Mismatched dsDNA substrate (target region shown in blue) mutation position 12 (red) | **GCTGCTCATGACAAATGAGATCGCAGGGCCGTTGTAAAGCGCCAGACTTG** |
| Trans-cleavage assay mismatch tolerance | Mismatched dsDNA substrate (target region shown in blue) mutation position 11 (red) | **GCTGCTCATGACAAATGAGATCGACGGGCCGTTGTAAAGCGCCAGACTTG** |
| Trans-cleavage assay mismatch tolerance | Mismatched dsDNA substrate (target region shown in blue) mutation position 10 (red) | **GCTGCTCATGACAAATGAGATCGAAAGGCCGTTGTAAAGCGCCAGACTTG** |
| Trans-cleavage assay mismatch tolerance | Mismatched dsDNA substrate (target region shown in blue) mutation position 9 (red) | **GCTGCTCATGACAAATGAGATCGAAGAGCCGTTGTAAAGCGCCAGACTTG** |
| Trans-cleavage assay mismatch tolerance | Mismatched dsDNA substrate (target region shown in blue) mutation position 8 (red) | **GCTGCTCATGACAAATGAGATCGAAGGACCGTTGTAAAGCGCCAGACTTG** |
| Trans-cleavage assay mismatch tolerance | Mismatched dsDNA substrate (target region shown in blue) mutation position 7 (red) | **GCTGCTCATGACAAATGAGATCGAAGGGACGTTGTAAAGCGCCAGACTTG** |
| Trans-cleavage assay mismatch tolerance | Mismatched dsDNA substrate (target region shown in blue) mutation position 6 (red) | **GCTGCTCATGACAAATGAGATCGAAGGGCAGTTGTAAAGCGCCAGACTTG** |
| Trans-cleavage assay mismatch tolerance | Mismatched dsDNA substrate (target region shown in blue) mutation position 5 (red) | **GCTGCTCATGACAAATGAGATCGAAGGGCCATTGTAAAGCGCCAGACTTG** |
| Trans-cleavage assay mismatch tolerance | Mismatched dsDNA substrate (target region shown in blue) mutation position 4 (red) | **GCTGCTCATGACAAATGAGATCGAAGGGCCGCTGTAAAGCGCCAGACTTG** |
| Trans-cleavage assay mismatch tolerance | Mismatched dsDNA substrate (target region shown in blue) mutation position 3 (red) | **GCTGCTCATGACAAATGAGATCGAAGGGCCGTCGTAAAGCGCCAGACTTG** |
| Trans-cleavage assay mismatch tolerance | Mismatched dsDNA substrate (target region shown in blue) mutation position 2 (red) | **GCTGCTCATGACAAATGAGATCGAAGGGCCGTTATAAAGCGCCAGACTTG** |
| Trans-cleavage assay mismatch tolerance | Mismatched dsDNA substrate (target region shown in blue) mutation position 1 (red) | **GCTGCTCATGACAAATGAGATCGAAGGGCCGTTGCAAAGCGCCAGACTTG** |

**Table S4. ssDNA oligonucleotide**

| **Purpose** | **Description** | **Sequence (5' 🡪 3')** |
| --- | --- | --- |
| Trans-cleavage assay target | Trans-cleavage ssDNA substrate with a length of 24 nt (target region shown in blue) | **ATGAGATCGAAGGGCCGTTGTAAA** |
| Trans-cleavage assay 5' end truncated target length | Trans-cleavage ssDNA substrate with a length of 9 nt (target region shown in blue) | **GGCCGTTGT** |
| Trans-cleavage assay 5' end truncated target length | Trans-cleavage ssDNA substrate with a length of 10 nt (target region shown in blue) | **GGGCCGTTGT** |
| Trans-cleavage assay 5' end truncated target length | Trans-cleavage ssDNA substrate with a length of 11 nt (target region shown in blue) | **AGGGCCGTTGT** |
| Trans-cleavage assay 5' end truncated target length | Trans-cleavage ssDNA substrate with a length of 12 nt (target region shown in blue) | **AAGGGCCGTTGT** |
| Trans-cleavage assay 5' end truncated target length | Trans-cleavage ssDNA substrate with a length of 13 nt (target region shown in blue) | **GAAGGGCCGTTGT** |
| Trans-cleavage assay 5' end truncated target length | Trans-cleavage ssDNA substrate with a length of 14 nt (target region shown in blue) | **CGAAGGGCCGTTGT** |
| Trans-cleavage assay 5' end truncated target length | Trans-cleavage ssDNA substrate with a length of 15 nt (target region shown in blue) | **TCGAAGGGCCGTTGT** |
| Trans-cleavage assay 5' end truncated target length | Trans-cleavage ssDNA substrate with a length of 16 nt (target region shown in blue) | **ATCGAAGGGCCGTTGT** |
| Trans-cleavage assay 5' end truncated target length | Trans-cleavage ssDNA substrate with a length of 17 nt (target region shown in blue) | **GATCGAAGGGCCGTTGT** |
| Trans-cleavage assay 5' end truncated target length | Trans-cleavage ssDNA substrate with a length of 18 nt (target region shown in blue) | **AGATCGAAGGGCCGTTGT** |
| Trans-cleavage assay 5' end truncated target length | Trans-cleavage ssDNA substrate with a length of 19 nt (target region shown in blue) | **GAGATCGAAGGGCCGTTGT** |
| Trans-cleavage assay 5' end truncated target length | Trans-cleavage ssDNA substrate with a length of 20 nt (target region shown in blue) | **TGAGATCGAAGGGCCGTTGT** |
| Trans-cleavage assay 3' end truncated target length | Trans-cleavage ssDNA substrate with a length of 9 nt (target region shown in blue) | **TGAGATCGA** |
| Trans-cleavage assay 3' end truncated target length | Trans-cleavage ssDNA substrate with a length of 10 nt (target region shown in blue) | **TGAGATCGAA** |
| Trans-cleavage assay 3' end truncated target length | Trans-cleavage ssDNA substrate with a length of 11 nt (target region shown in blue) | **TGAGATCGAAG** |
| Trans-cleavage assay 3' end truncated target length | Trans-cleavage ssDNA substrate with a length of 12 nt (target region shown in blue) | **TGAGATCGAAGG** |
| Trans-cleavage assay 3' end truncated target length | Trans-cleavage ssDNA substrate with a length of 13 nt (target region shown in blue) | **TGAGATCGAAGGG** |
| Trans-cleavage assay 3' end truncated target length | Trans-cleavage ssDNA substrate with a length of 14 nt (target region shown in blue) | **TGAGATCGAAGGGC** |
| Trans-cleavage assay 3' end truncated target length | Trans-cleavage ssDNA substrate with a length of 15 nt (target region shown in blue) | **TGAGATCGAAGGGCC** |
| Trans-cleavage assay 3' end truncated target length | Trans-cleavage ssDNA substrate with a length of 16 nt (target region shown in blue) | **TGAGATCGAAGGGCCG** |
| Trans-cleavage assay 3' end truncated target length | Trans-cleavage ssDNA substrate with a length of 17 nt (target region shown in blue) | **TGAGATCGAAGGGCCGT** |
| Trans-cleavage assay 3' end truncated target length | Trans-cleavage ssDNA substrate with a length of 18 nt (target region shown in blue) | **TGAGATCGAAGGGCCGTT** |
| Trans-cleavage assay 3' end truncated target length | Trans-cleavage ssDNA substrate with a length of 19 nt (target region shown in blue) | **TGAGATCGAAGGGCCGTTG** |
| Trans-cleavage assay 3' end truncated target length | Trans-cleavage ssDNA substrate with a length of 20 nt (target region shown in blue) | **TGAGATCGAAGGGCCGTTGT** |
| Trans-cleavage assay target length | Trans-cleavage ssDNA substrate with a length of 39 nt (target region shown in blue) | **TCATGACAAATGAGATCGAAGGGCCGTTGTAAAGCGCCA** |
| Trans-cleavage assay target length | Trans-cleavage ssDNA substrate with a length of 50 nt (target region shown in blue) | **GCTGCTCATGACAAATGAGATCGAAGGGCCGTTGTAAAGCGCCAGACTTG** |
| Trans-cleavage assay target length | Trans-cleavage ssDNA substrate with a length of 60 nt (target region shown in blue) | **GCTAGGCTGCTCATGACAAATGAGATCGAAGGGCCGTTGTAAAGCGCCAGACTTGTAAGC** |
| Trans-cleavage assay target length | Trans-cleavage ssDNA substrate with a length of 80 nt (target region shown in blue) | **TAATACGACTCACTATAGGGTCTAGGGACCGTGGCTCGAGCTCATGACAAATGAGATCGAAGGGCCGTTGTAAAGCGC** |
| Trans-cleavage assay mismatch tolerance | Mismatched ssDNA substrate (target region shown in blue) mutation position 20 (red) | **ACGAGATCGAAGGGCCGTTGTAAA** |
| Trans-cleavage assay mismatch tolerance | Mismatched ssDNA substrate mutation position 19 (red) | **ATAAGATCGAAGGGCCGTTGTAAA** |
| Trans-cleavage assay mismatch tolerance | Mismatched ssDNA substrate mutation position 18 (red) | **ATGCGATCGAAGGGCCGTTGTAAA** |
| Trans-cleavage assay mismatch tolerance | Mismatched ssDNA substrate mutation position 17 (red) | **ATGAAATCGAAGGGCCGTTGTAAA** |
| Trans-cleavage assay mismatch tolerance | Mismatched ssDNA substrate mutation position 16 (red) | **ATGAGCTCGAAGGGCCGTTGTAAA** |
| Trans-cleavage assay mismatch tolerance | Mismatched ssDNA substrate mutation position 15 (red) | **ATGAGACCGAAGGGCCGTTGTAAA** |
| Trans-cleavage assay mismatch tolerance | Mismatched ssDNA substrate mutation position 14 (red) | **ATGAGATAGAAGGGCCGTTGTAAA** |
| Trans-cleavage assay mismatch tolerance | Mismatched ssDNA substrate mutation position 13 (red) | **ATGAGATCAAAGGGCCGTTGTAAA** |
| Trans-cleavage assay mismatch tolerance | Mismatched ssDNA substrate mutation position 12 (red) | **ATGAGATCGCAGGGCCGTTGTAAA** |
| Trans-cleavage assay mismatch tolerance | Mismatched ssDNA substrate mutation position 11 (red) | **ATGAGATCGACGGGCCGTTGTAAA** |
| Trans-cleavage assay mismatch tolerance | Mismatched ssDNA substrate mutation position 10 (red) | **ATGAGATCGAAAGGCCGTTGTAAA** |
| Trans-cleavage assay mismatch tolerance | Mismatched ssDNA substrate mutation position 9 (red) | **ATGAGATCGAAGAGCCGTTGTAAA** |
| Trans-cleavage assay mismatch tolerance | Mismatched ssDNA substrate mutation position 8 (red) | **ATGAGATCGAAGGACCGTTGTAAA** |
| Trans-cleavage assay mismatch tolerance | Mismatched ssDNA substrate mutation position 7 (red) | **ATGAGATCGAAGGGACGTTGTAAA** |
| Trans-cleavage assay mismatch tolerance | Mismatched ssDNA substrate mutation position 6 (red) | **ATGAGATCGAAGGGCAGTTGTAAA** |
| Trans-cleavage assay mismatch tolerance | Mismatched ssDNA substrate mutation position 5 (red) | **ATGAGATCGAAGGGCCATTGTAAA** |
| Trans-cleavage assay mismatch tolerance | Mismatched ssDNA substrate mutation position 4 (red) | **ATGAGATCGAAGGGCCGCTGTAAA** |
| Trans-cleavage assay mismatch tolerance | Mismatched ssDNA substrate mutation position 3 (red) | **ATGAGATCGAAGGGCCGTCGTAAA** |
| Trans-cleavage assay mismatch tolerance | Mismatched ssDNA substrate mutation position 2 (red) | **ATGAGATCGAAGGGCCGTTATAAA** |
| Trans-cleavage assay mismatch tolerance | Mismatched ssDNA substrate mutation position 1 (red) | **ATGAGATCGAAGGGCCGTTGCAAA** |
| Trans-cleavage assay reporter | Trans-cleavage assay reporter ssDNA modified with FAM and BHQ1 | **FAM-TTATT-BHQ1** |

**Table S5. Oligonucleotides for EXP-J reaction**

| **Classification** | **Description** | **Sequence (5' 🡪 3')** |
| --- | --- | --- |
| miRNA | miR-21 | **UAGCUUAUCAGACUGAUGUUGA** |
| miRNA | miR-92a | **UAUUGCACUUGUCCCGGCCUGU** |
| miRNA | miR-222 | **AGCUACAUCUGGCUACUGGGU** |
| miRNA | miR-155 | **UUAAUGCUAAUCGUGAUAGGGGUU** |
| miRNA | miR-141 | **UAACACUGUCUGGUAAAGAUGG** |
| miRNA | miR-200c | **UAAUACUGCCGGGUAAUGAUGGA** |
| miRNA | let-7a | **UGAGGUAGUAGGUUGUAUAGUU** |
| miRNA | let-7f | **UGAGGUAGUAGAUUGUAUAGUU** |
| miRNA | miR-122 | **UGGAGUGUGACAAUGGUGUUUG** |
| miRNA | miR-345 | **GCUGACUCCUAGUCCAGGGCUC** |
| miRNA | miR-19a | **UGUGCAAAUCUAUGCAAAACUGA** |
| crRNA | Sequence contains 25 nt of the 3'-repeat fraction (blue) from the genomic Cas12j1 CRISPR array, followed by a 19 nt spacer corresponding to the target sequence | **AAACGAUUGCUCGAUUAGUCGAGACAUCAACUACUAUACUCUAA** |
| crRNA | Sequence contains 25 nt of the 3'-repeat fraction (blue) from the genomic Cas12j2 CRISPR array, followed by a 19 nt spacer corresponding to the target sequence | **CAACGAUUGCCCCUCACGAGGGGACAUCAACUACUAUACUCUAA** |
| crRNA | Sequence contains 25 nt of the 3'-repeat fraction (blue) from the genomic Cas12j3 RISPR array, followed by a 19 nt spacer corresponding to the target sequence | **UAUUGAUUGCCCAGUACGCUGGGACAUCAACUACUAUACUCUAA** |
| Trigger | 19 nt trigger (blue) | **TTAGAGTATAGTAGTTGAT** |
| Repeater | Repeater for 19 nt trigger (blue) | **ATCAACTACTATACTCTAAGTGAGACTCTATCAACTACTATACTCTAA–phosphate** |
| Repeater | Repeater for 18 nt trigger (blue) | **ATCAACTACTATACTCTAGTGAGACTCTATCAACTACTATACTCTA–phosphate** |
| Repeater | Repeater for 17 nt trigger (blue) | **ATCAACTACTATACTCTGTGAGACTCTATCAACTACTATACTCT–phosphate** |
| Repeater | Repeater for 16 nt trigger (blue) | **ATCAACTACTATACTCGTGAGACTCTATCAACTACTATACTC–phosphate** |
| Repeater | Repeater for 15 nt trigger (blue) | **ATCAACTACTATACTGTGAGACTCTATCAACTACTATAC–phosphate** |
| Converter | Converter for miR-21; Sequence contains trigger complementary fraction (blue), followed by NE recognition sequence (black) and miR-21-binding fraction (red) | **ATCAACTACTATACTCTAAGTGAGACTCTTCAACATCAGTCTGATAAGCTA–phosphate** |
| Converter | Converter for miR-92a; Sequence contains trigger complementary fraction (blue), followed by NE recognition sequence (black) and miR-92a-binding fraction (red) | **ATCAACTACTATACTCTAAGTGAGACTCTACCCAGTAGCCAGATGTAGCT–phosphate** |
| Converter | Converter for miR-222; Sequence contains trigger complementary fraction (blue), followed by NE recognition sequence (black) and miR-222-binding fraction (red) | **ATCAACTACTATACTCTAAGTGAGACTCTACAGGCCGGGACAAGTGCAATA–phosphate** |
| Converter | Converter for miR-155; Sequence contains trigger complementary fraction (blue), followed by NE recognition sequence (black) and miR-155-binding fraction (red) | **ATCAACTACTATACTCTAAGTGAGACTCTAACCCCTATCACGATTAGCATTAA–phosphate** |
| Reporter DNA | Reporter ssDNA modified with ROX and BHQ1 | **ROX–TTTTTTTT–BHQ1** |

**Table S6. Comparison of EXP-J reaction with previous CRISPR/Cas-based miRNA detection methods**

| **Method** | **Cas protein** | **crRNA length (nt)** | **Major components** | **Assay time** | **Detection limit** | **Detection sample** | **Reference** |
| --- | --- | --- | --- | --- | --- | --- | --- |
| RACE | Cas9 | 100 | 3 enzymes, 3 probes | 3.5 h | miR-21: 90 fM | Exosomal miRNA from cells, human plasma | S1 |
| Cas12a-SCR | LbaCas12a | 42 | 4 enzymes, 6 probes | 6 h | miR-21: 47 fM | Cell extracts | S2 |
| CRISPR-CRT |  | 41 | 2 enzymes, 4 probes | >2 h | miR-17: 1fM | Spiked serum | S3 |
| EXPCas |  | 41 | 3 enzymes, 3 probes | 40 min | miR-21: 103 fM | Spiked serum | S4 |
| SDA-Cas12a-hydrogel |  | 41 | 3 enzymes, 2 probes | 1.5 h | let-7a: 6.28 pM | Spiked serum, Cell extracts | S5 |
| CAL-LAMP |  | 68 | 3 enzymes, 6 probes | 1 h | let-7a: 0.1 fM | Cell extracts | S6 |
| PECL-CRISPR | LbuCas13a | 62 | 3 enzymes, 3 probes | 1.5 h | miR-17: 1 fM | Cell extracts | S7 |
| HyperCas |  | 62 | 4 enzymes, 4 probes | >3 h | miR-17: 0.2 fM | Cell extracts | S8 |
| CRISPR/Cas13a-mediated photoelectrochemical biosensors |  | 50 | 1 enzyme, 2 probes | >80 min | miR-21: 1 fM | Spiked serum, Cell extracts | S9 |
| Cas14SDA | Cas12f1  (Cas14) | 225 | 3 enzymes, 2 probes | 70 min | miR-21: 0.68 fM | Human blood | S10 |
| EDC-Cas14a |  | 170 | 1 enzyme, 5 probes | 2 h | miR-10b: 2.1 pM | Cell extracts | S11 |
| EXP-J | Cas12j3 | 44 | 3 enzymes, 3 probes | 40 min | miR-21: 1 fM | Cell extracts, human plasma | This work |

**Table S7. Oligonucleotides for qRT-PCR**

| **Purpose** | **Description** | **Sequence (5' 🡪 3')** |
| --- | --- | --- |
| Stem-loop qRT-PCR of miRNA | Stem-loop primer for miR-21 | **CTCAACTGGTGTCGTGGAGTCGGCAATTCAGTTGAGTCAACATC** |
| Stem-loop qRT-PCR of miRNA | Forward primer for miR-21 | **ACACTCCAGCTGGGTAGCTTATCAGACTGA** |
| Stem-loop qRT-PCR of miRNA | Stem-loop primer for miR-92a | **CTCAACTGGTGTCGTGGAGTCGGCAATTCAGTTGAGACAGGCCG** |
| Stem-loop qRT-PCR of miRNA | Forward primer for miR-92a | **ACACTCCAGCTGGGTATTGCACTTGTCCCG** |
| Stem-loop qRT-PCR of miRNA | Reverse primer | **CTCAACTGGTGTCGTGGAGTCGGCAA** |

**Table S8. Lung cancer patient information**

| **Sample number** | **Histologic type** | **Clinical stage** | **Gender** | **Age** |
| --- | --- | --- | --- | --- |
| 1 | Adenocarcinoma | Stage ⅢB | Female | 68 |
| 2 | Adenocarcinoma | Stage ⅣB | Male | 37 |
| 3 | Squamous cell carcinoma | Stage ⅡB | Male | 80 |
| 4 | Squamous cell carcinoma | Stage ⅢB | Female | 53 |
| 5 | Adenocarcinoma | Stage ⅠB | Male | 80 |
| 6 | Chronic necrotizing pulmonary aspergillosis | Stage ⅠB | Male | 58 |
| 7 | Squamous cell carcinoma | Stage ⅢB | Male | 67 |
| 8 | Collection of histiocytes with giant cell | Not available | Male | 69 |
| 9 | Small cell lung cancer + Squamous cell carcinoma | Stage ⅡB | Male | 82 |
| 10 | Squamous cell carcinoma | Stage ⅢB | Male | 68 |
| 11 | Adenocarcinoma | Stage ⅡB | Male | 48 |
| 12 | Squamous cell carcinoma | Stage ⅢB | Male | 72 |
| 13 | Acute and chronic inflammation with abscess | Not available | Female | 66 |
| 14 | Squamous cell carcinoma | Stage ⅠB | Male | 72 |
| 15 | Adenocarcinoma | Stage ⅡB | Male | 59 |
| 16 | Small cell carcinoma | Stage ⅢB | Male | 72 |
| 17 | Adenocarcinoma | Stage ⅣA | Female | 55 |
| 18 | Adenocarcinoma | Stage ⅡB | Male | 65 |
| 19 | No granuloma | Not available | Male | 51 |
| 20 | Adenocarcinoma | Stage ⅠB | Female | 64 |

**Table S9. Healthy control information**

| **Sample number** | **Histologic type** | **Clinical stage** | **Gender** | **Age** |
| --- | --- | --- | --- | --- |
| 1 | Not available | Not available | Male | 22 |
| 2 | Not available | Not available | Male | 46 |
| 3 | Not available | Not available | Male | 48 |
| 4 | Not available | Not available | Male | 47 |
| 5 | Not available | Not available | Male | 30 |
| 6 | Not available | Not available | Male | 48 |
| 7 | Not available | Not available | Male | 28 |
| 8 | Not available | Not available | Male | 55 |
| 9 | Not available | Not available | Male | 46 |
| 10 | Not available | Not available | Male | 41 |
| 11 | Not available | Not available | Male | 40 |
| 12 | Not available | Not available | Male | 24 |
| 13 | Not available | Not available | Male | 20 |
| 14 | Not available | Not available | Male | 35 |
| 15 | Not available | Not available | Male | 20 |
| 16 | Not available | Not available | Male | 20 |
| 17 | Not available | Not available | Male | 29 |
| 18 | Not available | Not available | Male | 38 |
| 19 | Not available | Not available | Male | 20 |
| 20 | Not available | Not available | Male | 39 |

**Table S10. Comparison of the characteristics of Cas proteins for diagnostic applications**

|  | **Cas9** | **Cas12a** | **Cas13a** | **Cas12f1**  **(Cas14)** | **Cas12j** |
| --- | --- | --- | --- | --- | --- |
| **Molecular**  **weight (kDa)** | 160-170 | 130-150 | 137-150 | 50-70 | 80-89 |
| **Oligomeric state** | Monomer | Monomer | Monomer | Dimer | Monomer |
| **Nuclease domain** | HNH and RuvC | RuvC | HEPN | RuvC | RuvC |
| **crRNA length (nt)** | 100^a^ | 40-44 | 62 | 140 ^a^ | 44 |
| **Target**  **Nucleic acid** | dsDNA | dsDNA | RNA | dsDNA | dsDNA |
| **Trans-cleavage**  **substrate** | No | ssDNA | RNA | ssDNA | ssDNA |
| **Protospacer Adjacent Motif (PAM)** | 5'-NGG-3' | 5'-TTTV-3' | - | 5'-TTN-3' | 5'-TTN-3' |
| **Yield of**  **purified protein** | ~4 mg/L | ~5 mg/L | ~1 mg/L | ~2 mg/L | ~25 mg/L |
| **Major diagnostic platform** | NASBACC | HOLMES/  DETECTR | SHERLOCK/ CARMEN | Cas14-DETECTR | EXP-J |
| **Diagnostic-linked amplification** | NASBA^b^ | RPA/LAMP  /RAA^c^ | NASBA/  RT-RPA^d^ | RPA | EXPAR |
| **Applications** | ZIKV^e^ | HPV^f^ | ZIKV/DENV^g^ | HER2 SNP^h^ | miRNA |
| **Reference** | S12 | S13 | S14, S15 | S16 | This work |
| a. The combined length of crRNA and tracrRNA  b. NASBA, Nucleic acid sequence-based amplification  c. RPA, recombinase polymerase amplification; LAMP, loop-mediated isothermal amplification; RAA, recombinase-aided amplification  d. RT-RPA, reverse transcription-RPA  e. ZIKV, Zika virus  f. HPV, human papillomavirus  g. DENV, dengue virus  f. HER2, human epidermal growth factor receptor 2; SNP, single-nucleotide polymorphism | | | | | |

**Reference**

S1. WANG, Ruixuan, et al. Rolling circular amplification (RCA)-Assisted CRISPR/Cas9 cleavage (RACE) for highly specific detection of multiple extracellular vesicle MicroRNAs. *Analytical chemistry*, 2019, 92.2: 2176-2185.

S2. WANG, Gaoting, et al. New CRISPR-derived microRNA sensing mechanism based on Cas12a self-powered and rolling circle transcription-unleashed real-time crRNA recruiting. *Analytical chemistry*, 2020, 92.9: 6702-6708.

S3. LONG, Xi, et al. CRISPR-Cas12a coupled with cyclic reverse transcription for amplified detection of miRNA. *Chemical Communications*, 2023, 59.50: 7763-7766.

S4. NIU, Chenqi, et al. CRISPR-Cas12a-assisted elimination of the non-specific signal from non-specific amplification in the Exponential Amplification Reaction. *Analytica Chimica Acta*, 2023, 1251: 340998.

S5. FENG, Shaoqiong, et al. Ultrasensitive Detection of miRNA via CRISPR/Cas12a Coupled with Strand Displacement Amplification Reaction. *ACS Applied Materials & Interfaces*, 2023.

S6. ZHANG, Mai, et al. CRISPR/Cas12a-assisted ligation-initiated loop-mediated isothermal amplification (CAL-LAMP) for highly specific detection of microRNAs. *Analytical Chemistry*, 2021, 93.22: 7942-7948.

S7. ZHOU, Ting, et al. CRISPR/Cas13a powered portable electrochemiluminescence chip for ultrasensitive and specific MiRNA detection. *Advanced Science*, 2020, 7.13: 1903661.

S8. HUANG, Mengqi, et al. Ultrasensitive and high-specific microRNA detection using hyper-branching rolling circle amplified CRISPR/Cas13a biosensor. *Sensors and Actuators B: Chemical*, 2020, 325: 128799.

S9. JIANG, Ling, et al. Ultrasensitive CRISPR/Cas13a-Mediated photoelectrochemical biosensors for specific and direct assay of miRNA-21. *Analytical Chemistry*, 2023, 95.2: 1193-1200.

S10. CHI, Zhen, et al. CRISPR-Cas14a-integrated strand displacement amplification for rapid and isothermal detection of cholangiocarcinoma associated circulating microRNAs. *Analytica Chimica Acta*, 2022, 1205: 339763.

S11. WANG, Xiaojuan, et al. A dual amplification strategy integrating entropy-driven circuit with Cas14a for sensitive detection of miRNA-10b. *Sensors and Actuators B: Chemical*, 2023, 397: 134666.

S12. PARDEE, Keith, et al. Rapid, low-cost detection of Zika virus using programmable biomolecular components. Cell, 2016, 165.5: 1255-1266.

S13. LI, Shi-Yuan, et al. CRISPR-Cas12a-assisted nucleic acid detection. Cell discovery, 2018, 4.1: 20.

S14. KELLNER, Max J., et al. SHERLOCK: nucleic acid detection with CRISPR nucleases. Nature protocols, 2019, 14.10: 2986-3012.

S15. ACKERMAN, Cheri M., et al. Massively multiplexed nucleic acid detection with Cas13. Nature, 2020, 582.7811: 277-282.

S16. HARRINGTON, Lucas B., et al. Programmed DNA destruction by miniature CRISPR-Cas14 enzymes. Science, 2018, 362.6416: 839-842.
